# Patch size drives colonization by aquatic insects, with minor priority effects of a cohabitant

**DOI:** 10.1002/ece3.8313

**Published:** 2021-11-13

**Authors:** Reed C. Scott, Matthew R. Pintar, William J. Resetarits

**Affiliations:** ^1^ Department of Biology Center for Water and Wetlands Resources Center for Biodiversity and Conservation Research University of Mississippi University Mississippi USA; ^2^ Institute of Environment Florida International University Miami Florida USA

**Keywords:** colonization, community assembly, habitat selection, patch size, priority effects, water beetles

## Abstract

Patch size is one of the most important factors affecting the distribution and abundance of species, and recent research has shown that patch size is an important niche dimension affecting community structure in aquatic insects. Building on this result, we examined the impact of patch size in conjunction with presence of larval anurans on colonization by aquatic insects. *Hyla chrysoscelis* (Cope's gray treefrog) larvae are abundant and early colonists in fishless lentic habitats, and these larvae can fill multiple ecological roles. By establishing larvae in mesocosms prior to colonization, we were able to assess whether *H*. *chrysoscelis* larvae have priority effects on aquatic insect assemblages. We conducted a series of three experiments in naturally colonized experimental landscapes to test whether (1) *H*. *chrysoscelis* larval density affects insect colonization, (2) variation in patch size affects insect colonization, and (3) the presence and larval density of *H*. *chrysoscelis* shift colonization of insects between patches of different size. Larval density independently had almost no effect on colonization, while patch size had species‐specific effects consistent with prior work. When larvae and patch size were tested in conjunction, patch size had numerous, often strong, species‐specific effects on colonization; larval density had effects largely limited to the assemblages of colonizing beetles and water bugs, with few effects on individual species. Higher larval densities in large mesocosms shifted some insect colonization to smaller patches, resulting in higher beta diversity among small patches in proximity to high density large mesocosms. This indicates establishing *H*. *chrysoscelis* larvae prior to insect colonization can likely create priority effects that slightly shape insect communities. Our results support the importance of patch size in studying species abundances and distributions and also indicate that colonization order plays an important role in determining the communities found within habitat patches.

## Graphical abstract

Patch size had strong effects on colonizing insect communities with some species preferring large patches and other species preferring small patches. Treefrog larvae have little direct effect on insect colonization, but when they had high densities in large patches, some of the insect colonization spilled over into adjacent small patches.

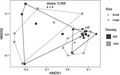

## 1 INTRODUCTION

Interspecific interactions influence metapopulation and metacommunity dynamics, determining whether patches are colonized by certain species and whether species can be sustained within a given patch (Hanski & Gilpin, 1991). In particular, priority effects suggest that the order in which species arrive can affect community assembly (Grainger & Gilbert, 2016). Priority effects are of two basic types, they can be stochastic such that the order of arrival of new species is variable over time or space, or they can be deterministic, where the order of arrival takes a consistent form based on phenology, life history, or habitat preferences, as in succession (Rudolf, 2019; Wilbur & Alford, 1985). These priority effects can either positively or negatively impact species richness within a metacommunity, depending on local adaptation and generation times of early colonists, as well as dispersal rates of later colonists (Vanoverbeke et al., 2016). If early colonists acclimate quickly to local conditions or late colonists have low dispersal rates, then the early colonists will dominate the metacommunity and decrease overall diversity (Grainger & Gilbert, 2016; Jones et al., 2020; Lawler & Morin, 1993; Shurin, 2001). If the reverse is true, or if early colonists are predators that relieve competitive exclusion effects, then early colonists can cause a net increase in species diversity (Beisner, 2001; McCauley & Briand, 1979; Sarnelle, 2005). Both predators and competitors can create priority effects (de Leeuw et al., 2020; Kennedy et al., 2009; Wilbur & Alford, 1985); however, a single species can fill the role of predator, prey, and competitor within a community. Therefore, when studying the mechanisms underlying priority effects, simply measuring changes in diversity does not necessarily capture all of the changes that may occur in communities. For instance, in communities with many different species, divergent responses by different species can generate unique community structures. Changes in component taxa and ecological guilds may help us better understand the overall impact that early colonists have on metacommunities.

Diversity and colonization rates are also affected by patch size; as patch size increases, there is a strong trend for species diversity and colonization rates to also increase (MacArthur & Wilson, 1963; Simberloff, 1974). Increased colonization is predicted by the target–area hypothesis: as patch size increases, colonization increases because larger patches have a higher probability of encounter (Gilpin & Diamond, 1976; Lomolino, 1990). This model assumes that larger patches only have increased immigration rates due to passive capture and that they are not being actively selected for by colonizing individuals. Two potential hypotheses explain the correlation between increased species diversity and increasing patch size. The first is the habitat diversity hypothesis, which suggests that larger patches have a greater diversity of habitat types, allowing for a greater diversity of species to survive within that patch (Williams, 1943). The second hypothesis, the stochastic extinction hypothesis, states that higher diversity in larger patches is due to increased immigration and decreased extinction (Connor & McCoy, 1979; Simberloff, 1974).

For some species, patch size can also act as a niche dimension—one of multiple environmental variables that determine their realized niche. Patch size is a major factor influencing colonization rates of insects in lentic freshwater systems; for instance, many aquatic heteropterans have strong preferences for larger patches, *Culex* mosquitoes prefer small patches, and aquatic beetles species‐specific preferences for large or small patches (Bohenek et al., 2017; Resetarits et al., 2019). Treefrogs (*Hyla chrysoscelis*) strongly prefer to oviposit in patches that have larger surface areas (Resetarits et al., 2018), deeper water (Pintar & Resetarits, 2017b), and that have recently filled (Pintar & Resetarits, 2017c), as many *Hyla* species are particularly sensitive to the presence of other taxa within a patch (Morin et al., 1990). The multitude of prior studies investigating development and habitat selection of *Hyla*, as well as colonization by aquatic insects, has utilized experimental mesocosms situated in natural landscapes that are particularly useful for testing ecological questions. Given that *H*. *chrysoscelis* are often among the earliest colonists of newly filled ponds, have abundant larvae, co‐occur with a variety of aquatic insects, and fill multiple ecological roles (competitors, prey), establishing *H*. *chrysoscelis* in experimental mesocosms prior to insect colonization mimics the natural colonization order and allows us to assay how one consistently early species can affect later colonization of a diverse insect assemblage.

We conducted a series of experiments in a naturally colonized experimental landscape to (1) determine whether colonizing insects respond to variation in larval *H*. *chrysoscelis* density while keeping patch size constant, (2) assess patch size preferences of colonizing insects, and (3) determine whether variation in density of *H*. *chrysoscelis* larvae within their preferred patch size (large) shifts colonization of insects among patches of different sizes.

## 2 MATERIALS AND METHODS

### 2.1 Study site and taxa

Our experiments were conducted at the University of Mississippi Field Station (UMFS) in Lafayette County, Mississippi, USA (34.4° N, 89.4° W), during the summer of 2019. At UMFS, there are 132 recorded species of aquatic beetles (Coleoptera) and 43 recorded species of aquatic and semiaquatic water bugs (Hemiptera: Heteroptera) (Pintar & Resetarits, 2020a, 2020b). With high abundances and numerous species co‐occurring in small habitat patches, aquatic insects are an ideal taxonomic group to study broad ecological questions. Aquatic insects can utilize a variety of aquatic habitats, from small temporary pools up to large peatlands (Batzer, 1996), and often select habitats based on predation, resource availability, canopy cover, and other patch characteristics (Binckley & Resetarits, 2007; Pintar & Resetarits, 2017a; Vonesh et al., 2009).

Most aquatic heteropterans are predaceous, with the primarily herbivorous Corixidae being an exception. Dytiscidae and Hydrophilidae are the dominant families of aquatic beetles, and both groups are predaceous during their larval stages. As adults, dytiscids are predaceous and hydrophilids are omnivorous scavengers, while haliplids are herbivores as larvae and adults (Short & White, 2019). Several taxa of both Coleoptera and Hemiptera are documented predators of larval anurans (Cronin & Travis, 1986). In particular, larvae of one dytiscid species in Australia, *Hydaticus parallelus*, are voracious predators of sandpaper frog (*Lechriodus fletcheri)* larvae, and adult *H*. *parallelus* preferentially oviposit in habitats containing *L*. *fletcheri* eggs, indicating that dytiscids may preferentially oviposit with and prey on amphibian larvae (Gould et al., 2019).

The larvae of *Hyla chrysoscelis* (Cope's gray treefrog; hereafter referred to as *Hyla*) occur in small temporary ponds, feed primarily on algae (Venesky et al., 2011), and metamorphose 3–6 weeks after hatching at UMFS (Pintar & Resetarits, 2017e). *Hyla* larvae, aquatic beetles, and Hemiptera commonly co‐occur, so it is likely that *Hyla* are both prey (for dytiscids, larval hydrophilids, notonectids, and other hemipterans) and competitors (for corixids, haliplids, and adult hydrophilids) (Morin et al., 1988). *Hyla* are abundant at UMFS and larvae can be easily reared, making them an ideal organism to test how presence of a single species that fulfills a variety of ecological roles can impact the abundance of species and community structure.

### 2.2 Experiment 1: *Hyla* larval density

The aim of this experiment was to determine whether colonization rates of aquatic insects are affected by the presence and density of *Hyla chrysoscelis* (hereafter *Hyla*) larvae while holding patch size constant. Mesocosms (~1200‐L cattle tanks: 2.54 m^2^, 1.80 m diameter) were randomly assigned one of five densities of *Hyla* larvae: 0, 75, 150, 300, or 600 larvae per mesocosm. Our maximum density was selected because (1) prior studies of *Hyla* development in similar mesocosms used densities up to 0.5 individuals per liter (Wilbur & Alford, 1985), (2) larval anuran development is highly density‐dependent with growth rates rapidly plateauing at very high densities (Wilbur, 1980, 1982), and (3) a prior experiment using mesocosms that allowed for freely colonizing and developing *Hyla* at the University of Mississippi Field Station (UMFS) had maximum observed densities around 600 individuals (W. J. Resetarits et al., unpublished data). Our lowest value represents a fraction of one female's clutch, while doubling densities allows us to test for possible density‐dependent responses by colonists.

**FIGURE 1:**
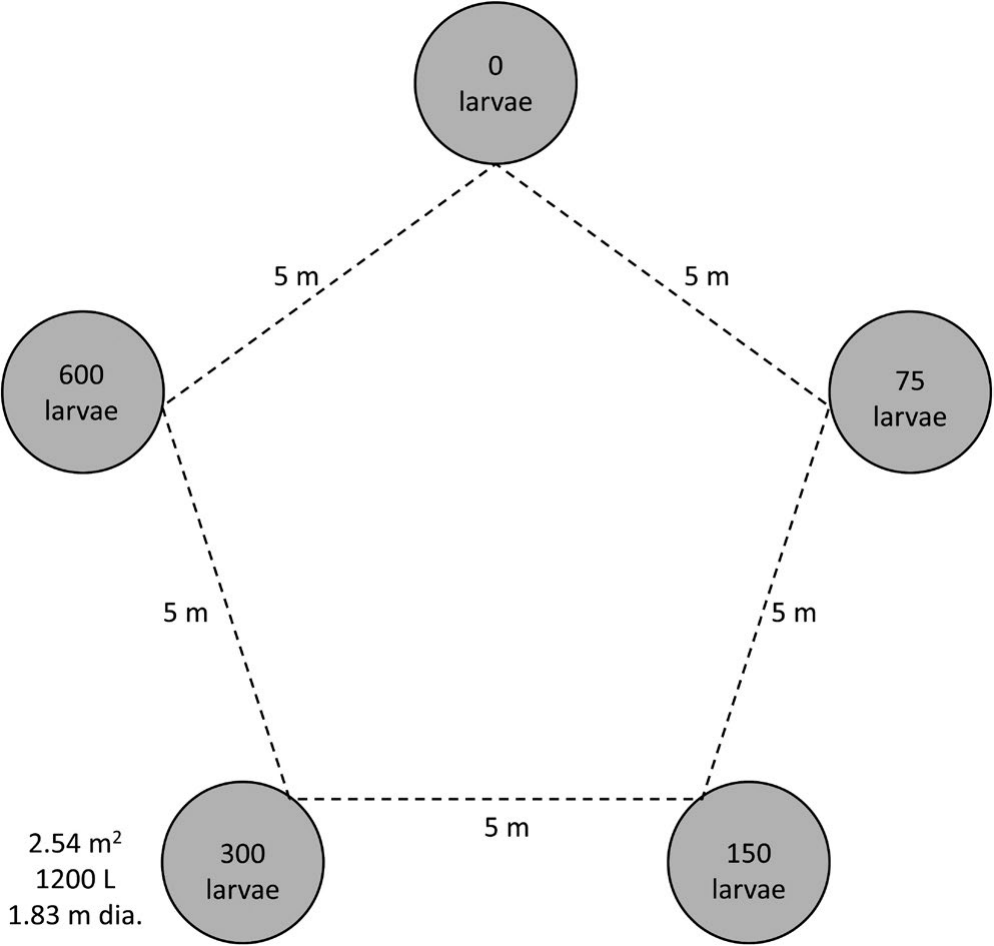
Layout of a block in the *Hyla* larval density experiment (exp. 1). Mesocosms were set up in a pentagon, with each tank being placed 5 m from adjacent tanks. Mesocosms were randomly assigned one of five different densities of *H*. *chrysoscelis* larvae (0, 75, 150, 300, 600 larvae per mesocosm).

*Hyla* eggs were collected from UMFS and larvae were raised to the 17th–20th Gosner stage (Gosner, 1960) in separate rearing mesocosms prior to addition to experimental mesocosms. Using a randomized complete block design, mesocosms were arranged in a pentagonal shape (Figure 1) within each of the 9 blocks (*N* = 45), with each mesocosm placed 5 m from adjacent mesocosms. Mesocosms were filled with well water, 2.2 kg of hardwood leaf litter, 2 L of pond inoculum (fishless pond water) to simulate microbial, planktonic, and algal communities, and *Hyla* larvae corresponding to one of the five density treatments. Mesocosms were then covered with 1.3 × 1.13 mm mesh screens, which were sunk to allow for colonization above the screens and separation of colonists and *Hyla* larvae. Three blocks were set up at one time, so the block effect had both a spatial and temporal component. Blocks were run for 3 weeks before being terminated to reduce potential effects from changing larval densities via metamorphosis (Pintar & Resetarits, 2017e). Between each round, mesocosms were drained and cleaned, while all tadpoles and leaf litter were deposited into nearby ponds. We ran three rounds of the experiment with blocks 1–3 run from May 28 to June 18, blocks 4–6 from June 28 to July 19, and blocks 7–9 from July 31 to August 21. Once screens were submerged and colonization began, adult insects were collected weekly, preserved, identified, and quantified. Insect identifications followed Pintar and Resetarits (2020a, 2020b), with most taxa identified to species and some only to genus.

Here, because patch size did not vary, we assessed effects on square‐root‐transformed raw abundances of colonizing insects. Our primary response variables are five community (assemblage) metrics: abundance of all insects, taxonomic richness, alpha diversity (Jost's effective number of species [inverse Simpson]; Jost, 2006), assemblage structure (PERMANOVA), and beta diversity (distance to median). The richness analysis included overall insect abundance as a covariate, as the two are expected to positively covary. We did not necessarily expect equivalent colonization patterns among different taxa, and individual taxa are largely expected to be independent (Pintar & Resetarits, 2020c), so we also separately analyzed individual taxa with abundances >100. Abundances of all taxa and richness were summed across the duration of the experiment and square‐root‐transformed ($\sqrt{X+0.5}$). For univariate analyses, we used linear mixed‐effects models fit by maximum likelihood using the Satterthwaite method with type III sums of squares to analyze the effect of treatment variables as fixed effects with block variables as a random effects on square‐root‐transformed data using the lme4 package v 1.1‐27.1, lmerTest package v 3.1‐3, and multcomp package v 1.4‐17 in R v 4.1.1 (Bates et al., 2015; Hothorn et al., 2008, Kuznetsova et al., 2017; R Core Team, 2021). Insect assemblages were analyzed with PERMANOVA (adonis; square‐root‐transformed; Bray–Curtis distances) to test for differences in multivariate centroid location (average assemblage composition) and betadisper to examine differences in multivariate dispersion (beta diversity; distance to median) with the vegan package v 2.5‐7 (Oksanen et al., 2017). Assemblage structure was visualized with non‐metric multidimensional scaling (NMDS). We set *α* = 0.05 for all analyses and include estimated effect sizes ($\eta_{P}^{2}$).

Mixed‐effects univariate analyses included treatment (larval *Hyla* density) as a categorical fixed effect with location (spatial position at UMFS) and round (time) as random effects. Blocks were placed at the same locations in each round, so location and round better capture spatial and temporal variation, respectively, than a single block random effect. Significant main effects of *Hyla* density in univariate analyses were followed up with Dunnett's procedure to compare all individual *Hyla* densities to controls. In the assemblage structure (PERMANOVA) analysis, *Hyla* density, location, and round were all fixed main effects due to limitations of accounting for multiple random effects.

### 2.3 Experiment 2: Patch size

**FIGURE 2:**
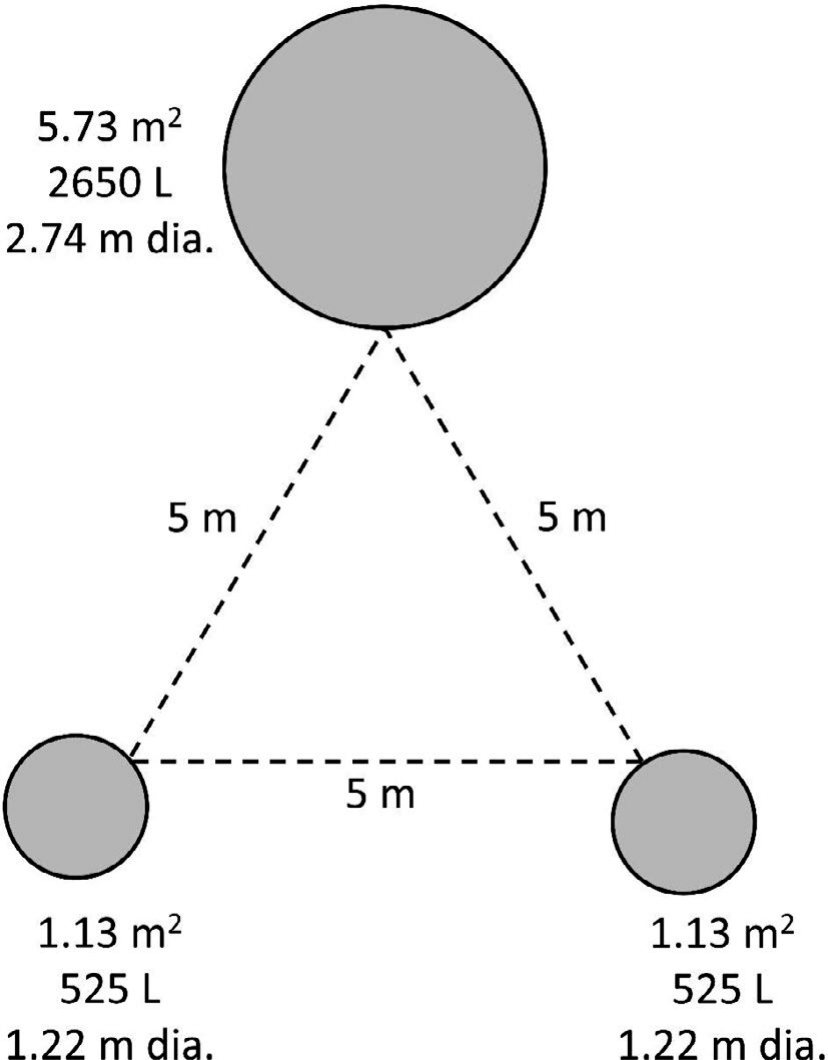
Design of one block (locality) for both the patch size (exp. 2) and *Hyla chrysoscelis* larval density + patch size (exp. 3) experiments, with one large and two small mesocosms arranged in an equilateral triangle and separated by 5 m. Each block was 5 m from the forest edge, measured from the closest point. Each block was ≥10 m from other blocks. Figure is drawn approximately to scale. In experiment 2, no larval *H. chrysoscelis* were added to mesocosms. In experiment 3, *H*. *chrysoscelis* larvae were added to large mesocosms at two different densities: either 600 or 1200 larvae; small mesocosms did not receive any larvae

Our objective in this experiment was to assess how patch size affects abundances and assemblage structure of aquatic insects. This experiment was conducted to independently test the effects of patch size using the same physical layout as experiment 3 below because the layout of these experiments were different from that tested by Resetarits et al. (2019), who were the first to report differences in insect colonization across patches of different size. Six blocks were constructed across two fields at UMFS, and each block contained three mesocosms of two different sizes: one large mesocosm (5.73 m^2^ diameter) and two small mesocosms (1.13 m^2^ diameter). Mesocosms within the same block were set up in an equilateral triangle and placed 5 m from each other (Figure 2), and blocks were set up 5 m from the forest's edge and ≥10 m from all other blocks.

Within a block, placement of mesocosms was randomly assigned (cardinal direction of small mesocosms around large mesocosm). Once mesocosms were arranged on July 14, 2019, they were filled to a depth of 50 cm such that large mesocosms held ~2650 L and small pools had ~525 L. Large and small mesocosms had 4.4 or 0.9 kg of hardwood leaf litter added, respectively, along with 4 L or 1 L of pond inoculum. Once mesocosms were filled they were covered with tight fitting fiberglass screen lids (1.3 × 1.13 mm) to prevent colonization. On July 15, after all mesocosms were filled, screens were sunk, and the experiment began. Insects were collected and preserved weekly, and later quantified and identified following Pintar and Resetarits (2020a, 2020b). The experiment was terminated on August 20, 2019.

Because our aim here was to determine whether colonization rates varied relative to patch size, our univariate response variables were the square‐root‐transformed area‐adjusted colonization rates (# individuals/patch area) of colonizing insects. Richness, alpha and beta diversity, and abundances in multivariate analyses were not adjusted relative to patch area. Mixed‐effects univariate and PERMANOVAs included patch size as a fixed effect with block as a random effect. The same set of analyses were performed as in experiment 1.

### 2.4 Experiment 3: *Hyla* larval density + patch size

The objectives of our third experiment were to (1) verify patch size preferences of colonizing insects and (2) determine whether higher densities of *Hyla* larvae that occur in their preferred patches (large) shifted colonization of insects among patches within the same locality. We established localities (blocks) of three mesocosms, with a total of 12 blocks (36 total mesocosms). Each block consisted of 1 large (5.73 m^2^, 2.74 m diameter, ~2650 L) and 2 small mesocosms (1.13 m^2^, 1.22 m diameter, ~525 L) (Figure 2). We manipulated the density of *Hyla* larvae added to the large mesocosm (600 or 1200 larvae per large mesocosm), while the small mesocosms did not receive any larvae. Therefore, while variation in *Hyla* density is applied to a single mesocosm, it is a locality‐level treatment since patch size and *Hyla* density are not truly crossed, as the density treatment is only applied to large mesocosms. Thus, we present the choice of two patch types, large with larvae and small without larvae, and two locality types, with either 1200 larvae or 600 larvae in large patches.

Mesocosms within the same block were set up in an equilateral triangle and placed 5 m from each other (Figure 2). Blocks were set up 5 m from the forest's edge and ≥10 m from all other blocks. Within a block, placement of mesocosms was randomly assigned (cardinal direction of small mesocosms around large mesocosm). Once mesocosms were arranged, they were filled to a depth of 50 cm, with 4.4 kg or 0.9 kg of hardwood leaf litter and 4 L or 1 L of pond inoculum added to large and small mesocosms, respectively. After mesocosms were filled, they were covered with tight fitting fiberglass screen lids (1.3 × 1.13 mm) to prevent colonization.

Prior to setting up this experiment, we collected *H*. *chrysoscelis* eggs and raised them to Gosner stage 17–20 (Gosner, 1960). Once enough larvae were reared, they were added to the large mesocosms and screens were submerged to allow colonization. Colonizing insects were collected weekly, preserved, and later identified following Pintar and Resetarits (2020a, 2020b). Mesocosms were checked daily for newly oviposited *Hyla* eggs, which were then removed to maintain initial densities within mesocosms.

All blocks were taken down after three weeks in order to avoid loss of larvae due to metamorphosis, and only six blocks were set up at one time. Mesocosms were drained and cleaned between each round, and all larvae and leaf litter were deposited in nearby ponds. As such, there were two rounds of the experiment, with blocks 1–6 running from May 24–June 15 and blocks 7–12 running from June 20 to July 11. There were three replicates of each density in each round, and density treatments were randomly assigned to blocks in both rounds. Thus, there were a total of six replicates of each locality treatment (1200 vs. 600).

In analyses, we separately assessed the effects of patch size and *Hyla* density because (1) *Hyla* density was treated as a locality‐level treatment, as variation in larval density was only applied to large mesocosms (1 per locality) and hence only varied between large mesocosms in different localities (blocks), (2) there was ≥10 m spatial separation between blocks, and (3) abundances of dispersing insects can be highly localized. All three of these factors mean that location effects can be confounded with locality‐level treatment effects (*Hyla* density). Although patch size and presence of *Hyla* larvae are confounded in this experiment, the consistently strong effect of patch size (Resetarits et al., 2019; experiment 2 [see results]) and the lack of effects of *Hyla* larvae on insect colonization (experiment 1; see results) suggest our test between large and small patches here is representative of patch size effects and not *Hyla* presence/absence. Separate assessment of patch size here can be useful because there is typically variation in abundance and composition of species across space and time even on small scales, such as across the UMFS, allowing us to potentially capture responses by additional species.

The same series of analyses were conducted as outlined in experiment 1. Analysis of alpha diversity was a single analysis for the entire experimental design (alpha diversity = size + density), as it is an index that cannot be partitioned like our raw data. For abundance (overall and individual taxa), richness, assemblage structure, and beta diversity, analyses followed a two‐tiered approach: first analyzing effects of patch size and then effects of density (separate analyses). In the analysis of taxonomic richness, we assessed the effects of patch size through a mixed‐effects model on the square‐root‐transformed number of taxa with overall insect abundance as a covariate, as richness and abundances are expected to positively covary. We then used logistic regression to assess effects of *Hyla* density separately among small and large patches by asking whether the proportion of taxa within localities that colonized a patch varied based on *Hyla* density; a single species can occur in multiple patches and so the number of species per patch cannot be directly compared among mesocosms for density analyses. In assemblage structure and beta diversity analyses, we assessed the square‐root‐transformed abundances that were unadjusted for patch size. For both assemblage structure and beta diversity, we first assessed the effects of patch size on the full experimental design (*N* = 36) and then assessed the effects of *Hyla* density among large mesocosms (*N* = 12) and among small mesocosms (*N* = 24).

To determine effects on abundance of all insects and common taxa (*N* > 100), we first assessed how the area‐adjusted colonization rate (# individuals/patch area; square‐root‐transformed) varied based on patch size. These mixed‐effects univariate analyses included patch size as a fixed effect with location and round (time) as random effects. We then assessed the effects of *Hyla* density by asking if the proportion of colonists within localities that colonized large patches varied based on the locality‐level treatment (*Hyla* density). We used logistic regression with block nested within round, and location as random effects and a binomial distribution to assess whether this proportion varied based on *Hyla* density for both abundance and richness analyses (Warton & Hui, 2011).

## 3 RESULTS

### 3.1 Experiment 1: *Hyla* larval density

**FIGURE 3:**
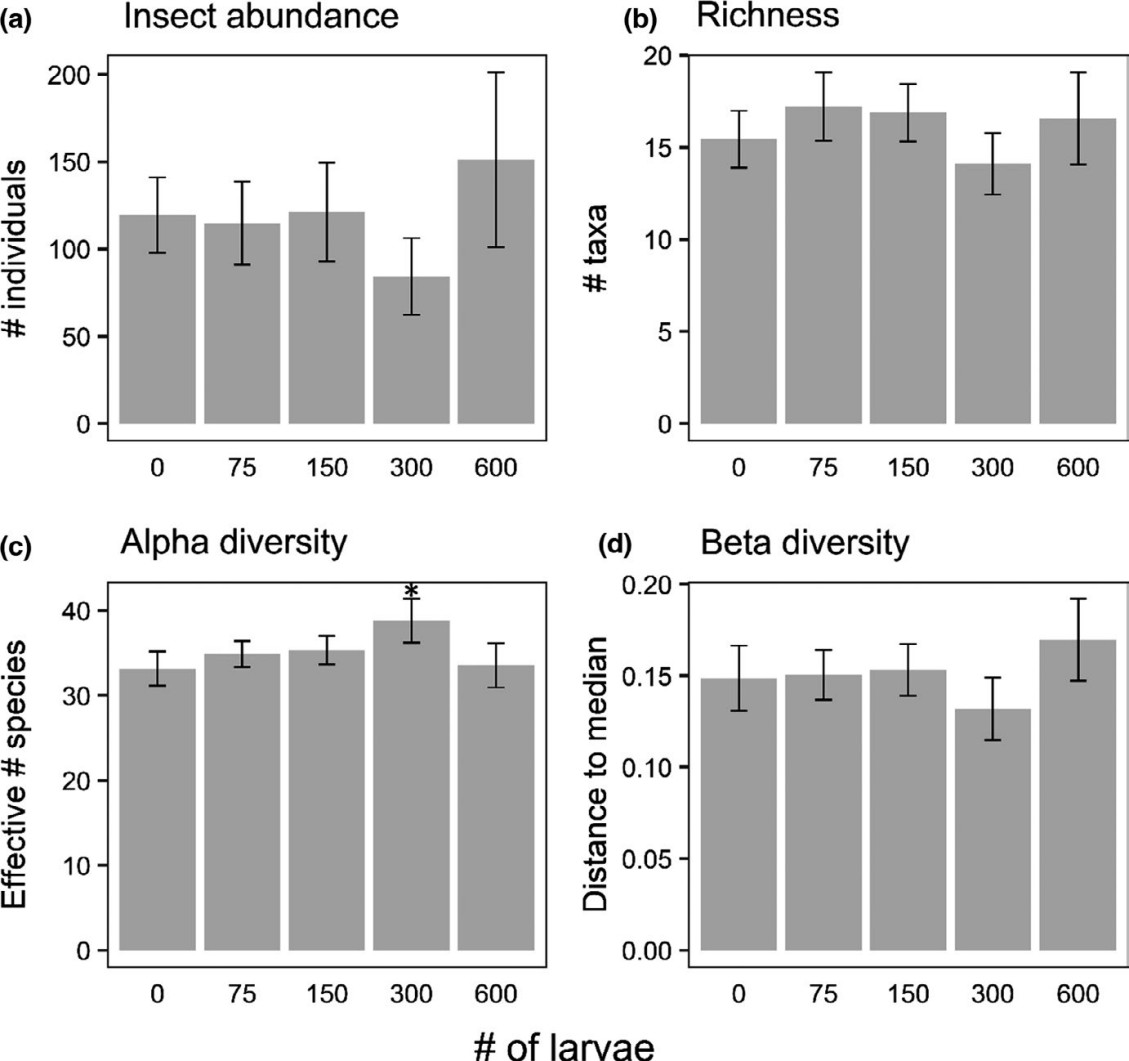
Mean (± *SE*) insect assemblage results across the five densities of larval *H*. *chrysoscelis* in the *Hyla* larval density experiment (exp. 1): (a) abundance of all insects, (b) taxonomic richness, (c) alpha diversity (Jost's effective number of species), and (d) beta diversity (distance to median). The asterisk indicates a significant difference (*p* < .05) from the control (0 larvae) with Dunnett's procedure. No other comparisons to controls were significant

**FIGURE 4:**
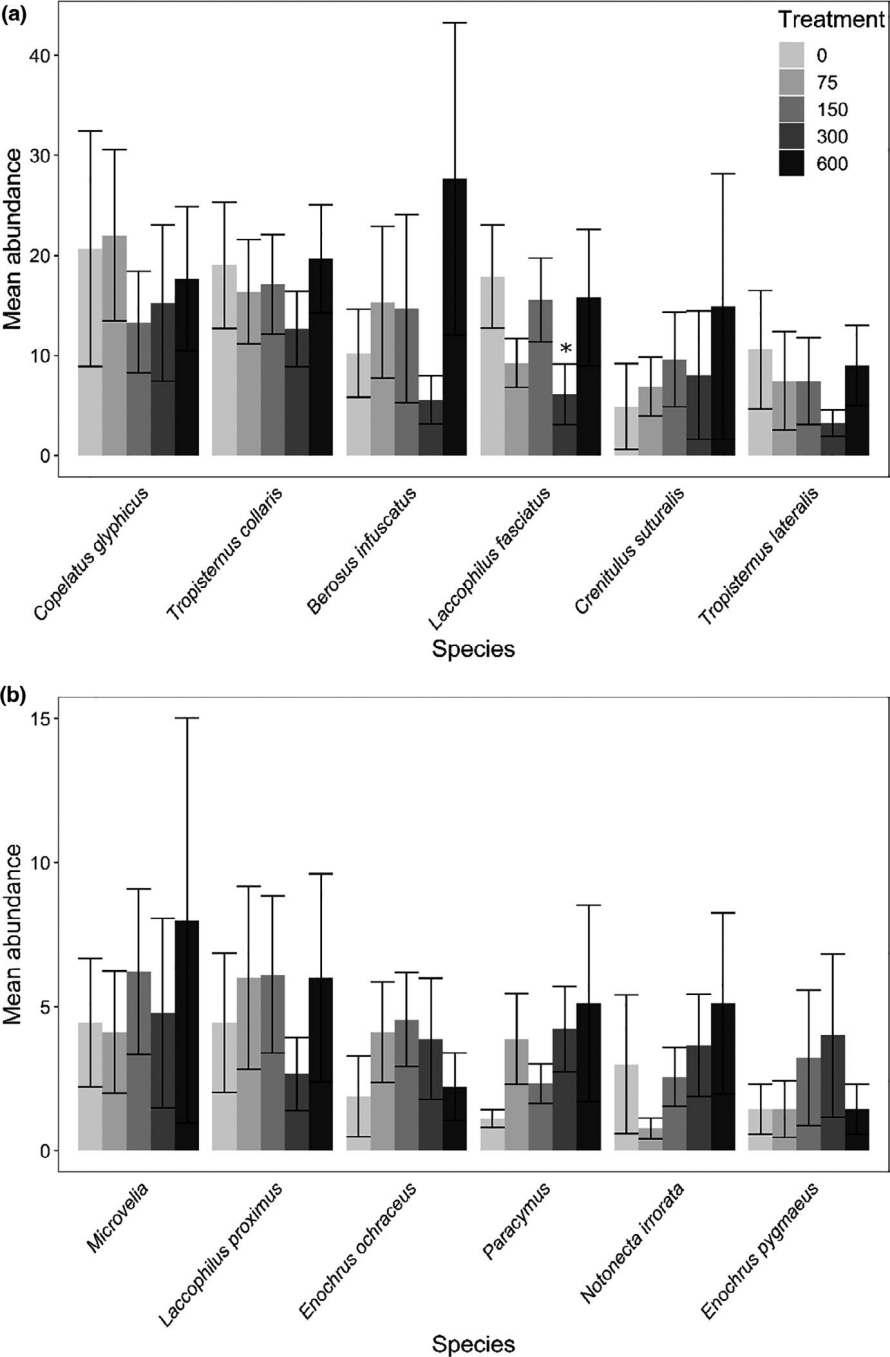
Average number of colonists (± *SE*) for the 12 most abundant taxa (*N* > 100) across the five densities of larval *H*. *chrysoscelis* in the *Hyla* larval density experiment (exp. 1). Taxa are listed in order of abundance in (a) and then (b) (see Tables 1 and 2). For *Laccophilus fasciatus*, the asterisk indicates a significant difference (*p* < .05) from the control (0 larvae) determined with Dunnett's procedure. No other taxa had significant main effects of *Hyla* density in analyses

**FIGURE 5:**
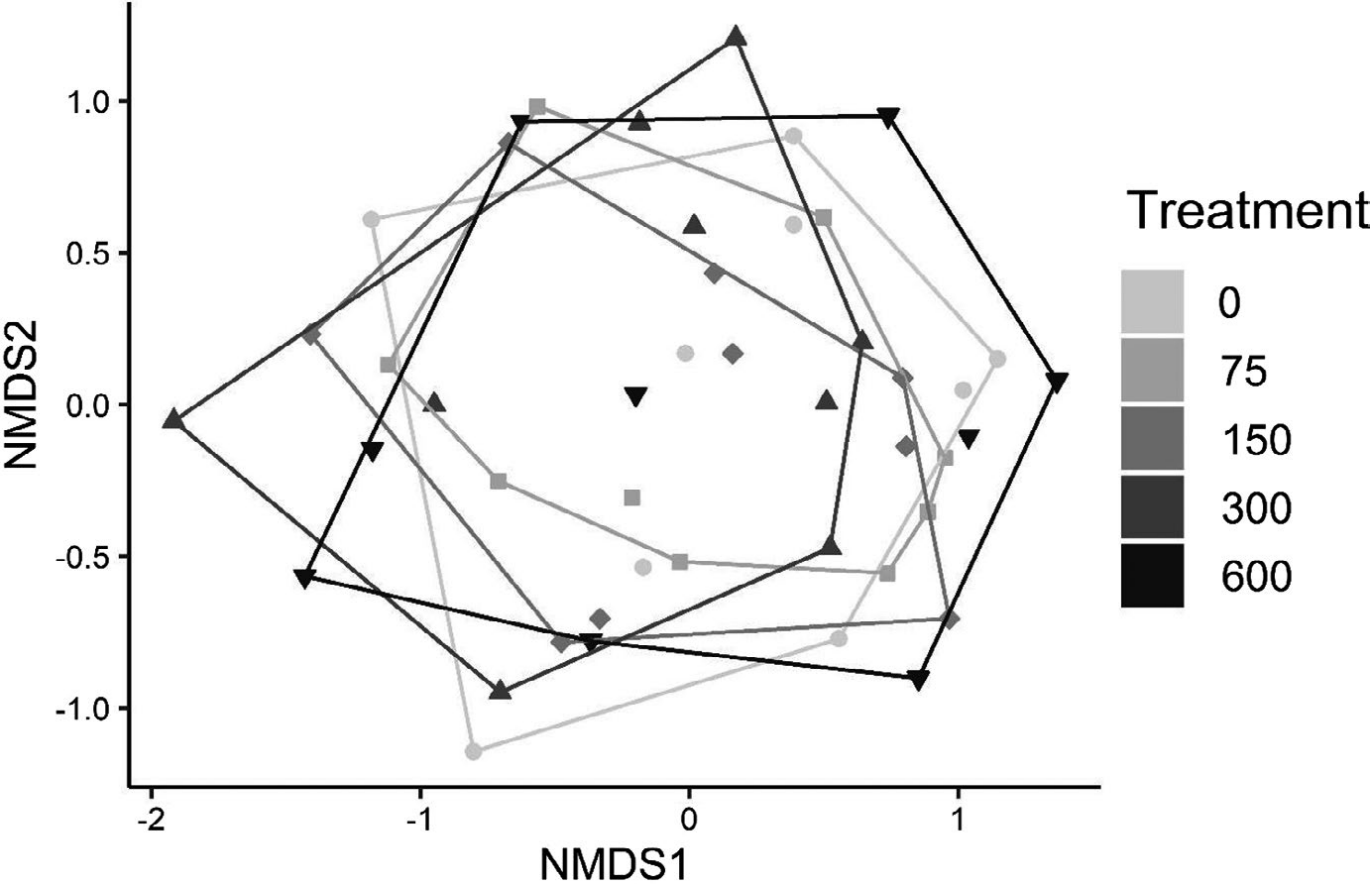
NMDS plots for the insect assemblage in the *Hyla* larval density experiment (exp. 1) across the five larval *H*. *chrysoscelis* densities (treatment)

A total of 4702 beetles representing 47 species/genera in seven families (Table 1) and 613 hemipterans representing 12 species/genera in 6 families colonized the experiment (Table 2). There were very few responses to variation in larval *Hyla* density by colonizing insects, as shown by most community metrics and 11 of the 12 most abundant taxa (Table 3, Figures 3, 4). Although there were no differences in overall insect abundance (Figure 3a), richness (Figure 3b), assemblage structure (Figure 5), or beta diversity (Figure 3d), there was a marginal effect on alpha diversity (Figure 3c), with Dunnett's procedure indicating mesocosms with 300 larvae having a higher effective number of species than the controls (0 larvae). The only species with an effect of *Hyla* density was *Laccophilus fasciatus*, which had fewer colonists in mesocosms with 300 larvae than controls, and marginally fewer in mesocosms with 75 larvae than controls (Figure 4a). As expected, richness strongly positively covaried with total insect abundance (Table 3). Assemblage structure had significant effects of location and round, while these blocking factors were random effects in other models.

**TABLE 1:** Taxa and abundances of colonizing Coleoptera (beetles) in our three experiments. Density represents the abundances in the *Hyla* larval density experiment (exp. 1); size indicates the abundances in the patch size experiment (exp. 2), and D+S indicates the abundances in the *Hyla* larval density + patch size experiment (exp. 3). Bold indicates family lines are sums of all species/genera within each family

Taxon	Density	Size	D+S	Taxon	Density	Size	D+S
**Dytiscidae**	**1780**	**524**	**1939**	**Hydraenidae**	**7**	**0**	**7**
*Acilius fraternus*	1	2	13	*Hydraena marginicollis*	7	0	7
*Acilius mediatus*	2	1	4	**Hydrochidae**	**8**	**0**	**2**
*Bidessonotus inconspicuus*	6	1	5	*Hydrochus* spp.	1	0	2
*Celina hubbelli*	3	0	8	*Hydrochus rugosus*	7	0	0
*Copelatus glyphicus*	800	223	1020	**Hydrophilidae**	**2834**	**404**	**1891**
*Copelatus chevrolati*	36	13	25	*Berosus aculeatus*	19	0	4
*Coptotomus venustus*	3	0	0	*Berosus exiguus*	35	0	4
*Desmopachria*	1	0	0	*Berosus infuscatus*	661	32	77
*Hydaticus bimarginatus*	17	20	32	*Berosus pantherinus*	2	0	0
*Hydrocolus deflatus*	0	0	1	*Berosus peregrinus*	3	0	1
*Hydrocolus oblitus*	0	0	1	*Berosus pugnax*	11	0	0
*Hydroporus pseudoniger*	0	0	1	*Berosus sayi*	61	2	15
*Hydroporus rufilabris*	12	0	32	*Crenitulus suturalis*	398	3	560
*Laccophilus fasciatus*	581	157	647	*Cymbiodyta chamberlaini*	22	1	70
*Laccophilus proximus*	227	85	75	*Derallus altus*	1	0	1
*Meridiorhantus calidus*	17	12	9	*Enochrus blatchleyi*	4	0	3
*Neobidessus pullus*	6	0	0	*Enochrus cinctus*	0	0	5
*Neoporus blanchardi*	0	0	23	*Enochrus consors*	3	0	0
*Neoporus undulatus*	0	0	3	*Enochrus consortus*	0	0	2
*Thermonectus basillaris*	44	7	18	*Enochrus fimbriatus*	8	3	17
*Uvarus granarius*	7	1	2	*Enochrus ochraceus*	150	77	484
*Uvarus lacustris*	17	2	20	*Enochrus pygmaeus*	104	3	4
**Elmidae**	**1**	**1**	**0**	*Helochares maculicollis*	21	3	25
*Dubiraphia minima*	1	0	0	*Hydrochara soror*	16	26	49
*Stenelmis sinuata*	0	1	0	*Hydrochara spangleri*	2	2	3
**Haliplidae**	**69**	**2**	**232**	*Paracymus* spp.	150	18	248
*Haliplus triopsis*	2	0	0	*Tropisternus blatchleyi*	58	8	32
*Peltodytes muticus*	67	2	232	*Tropisternus collaris*	763	157	193
**Helophoridae**	**3**	**0**	**5**	*Tropisternus lateralis*	339	68	94
*Helophorus linearis*	3	0	2	*Tropisternus natator*	3	1	0
*Helophorus lineatus*	0	0	3				

**TABLE 2:** Taxa abundances of colonizing Hemiptera (true bugs; Heteroptera) in our three experiments. Density represents the abundances in the *Hyla* larval density experiment (exp. 1); Size indicates the abundances in the patch size experiment (exp. 2), and D+S indicates the abundances in the *Hyla* larval density + patch size experiment (exp. 3). Bold indicates infraorder and family lines are sums of all species/genera within each respective taxon

Taxon	Density	Size	D+S
**gerromorpha**	**271**	**30**	**322**
**Gerridae**	**21**	**6**	**63**
*Gerris argenticollis*	1	0	1
*Gerris marginatus*	0	0	2
*Limnoporus canaliculatus*	20	6	60
**Hebridae**	**1**	**0**	**0**
*Hebrus burmeisteri*	1	0	0
**Mesoveliidae**	**1**	**1**	**0**
*Mesovelia amoena*	0	1	0
*Mesovelia mulsanti*	1	0	0
**Veliidae**	**248**	**23**	**259**
*Microvelia* sp. 1	0	0	1
*Microvelia* spp.	248	23	258
**nepomorpha**	**342**	**218**	**1178**
**Corixidae**	**204**	**71**	**548**
*Hesperocorixa* spp.	72	52	535
*Hesperocorixa minor*	2	4	1
*Sigara* spp.	22	11	11
*Trichocorixa calva*	81	4	1
*Trichocorixa kanza*	27	0	0
**Nepidae**	**0**	**0**	**3**
*Ranatra buenoi*	0	0	3
**Notonectidae**	**138**	**147**	**627**
*Buenoa* spp.	2	2	11
*Notonecta indica*	0	0	1
*Notonecta irrorata*	136	145	615

**TABLE 3:** Analysis results from the *Hyla* larval density experiment (exp. 1). All results are for the effects of larval *H*. *chrysoscelis* density, with the exception of richness and assemblage structure, which include additional factors in analyses (listed individually below). $\eta_{P}^{2}$ is an estimate of effect size. Bold indicates significant results (*p* < .05)

	SS	*df*	*F*	*p*	$\eta_{P}^{2}$
Insect abundance	34.37	4, 39.9	1.7	.16	0.16
Richness					
Abundance	11.26	1, 44.7	90.3	**<.0001**	0.68
Density	0.48	4, 42.1	1.0	.44	0.08
Alpha diversity	178.10	4, 39.9	2.2	.09	0.18
Assemblage structure (PERMANOVA)					
Density	0.06	4, 36	1.3	.20	0.05
Location	0.41	2, 36	16.4	.**0001**	0.26
Round	0.26	2, 36	10.5	.**0001**	0.18
Beta diversity	0.01	4, 40	0.6	.66	0.06
*Berosus infuscatus*	13.37	4, 39.9	1.4	.26	0.12
*Copelatus glyphicus*	4.42	4, 39.8	0.3	.86	0.03
*Crenitulus suturalis*	5.32	4, 39.9	0.7	.60	0.07
*Enochrus ochraceus*	3.22	4, 39.5	1.0	.44	0.08
*Enochrus pygmaeus*	1.00	4, 39.9	0.5	.75	0.05
*Laccophilus fasciatus*	17.81	4, 39.9	5.2	.**0017**	0.34
*Laccophilus proximus*	2.15	4, 40.0	1.0	.40	0.09
*Microvelia*	1.25	4, 39.9	0.3	.85	0.03
*Notonecta irrorata*	2.78	4, 39.9	1.0	.43	0.10
*Paracymus*	3.22	4, 39.2	1.0	.43	0.09
*Tropisternus collaris*	3.07	4, 40.0	0.8	.52	0.08
*Tropisternus lateralis*	5.32	4, 39.9	1.2	.31	0.11

### 3.2 Experiment 2: Patch size

**FIGURE 6:**
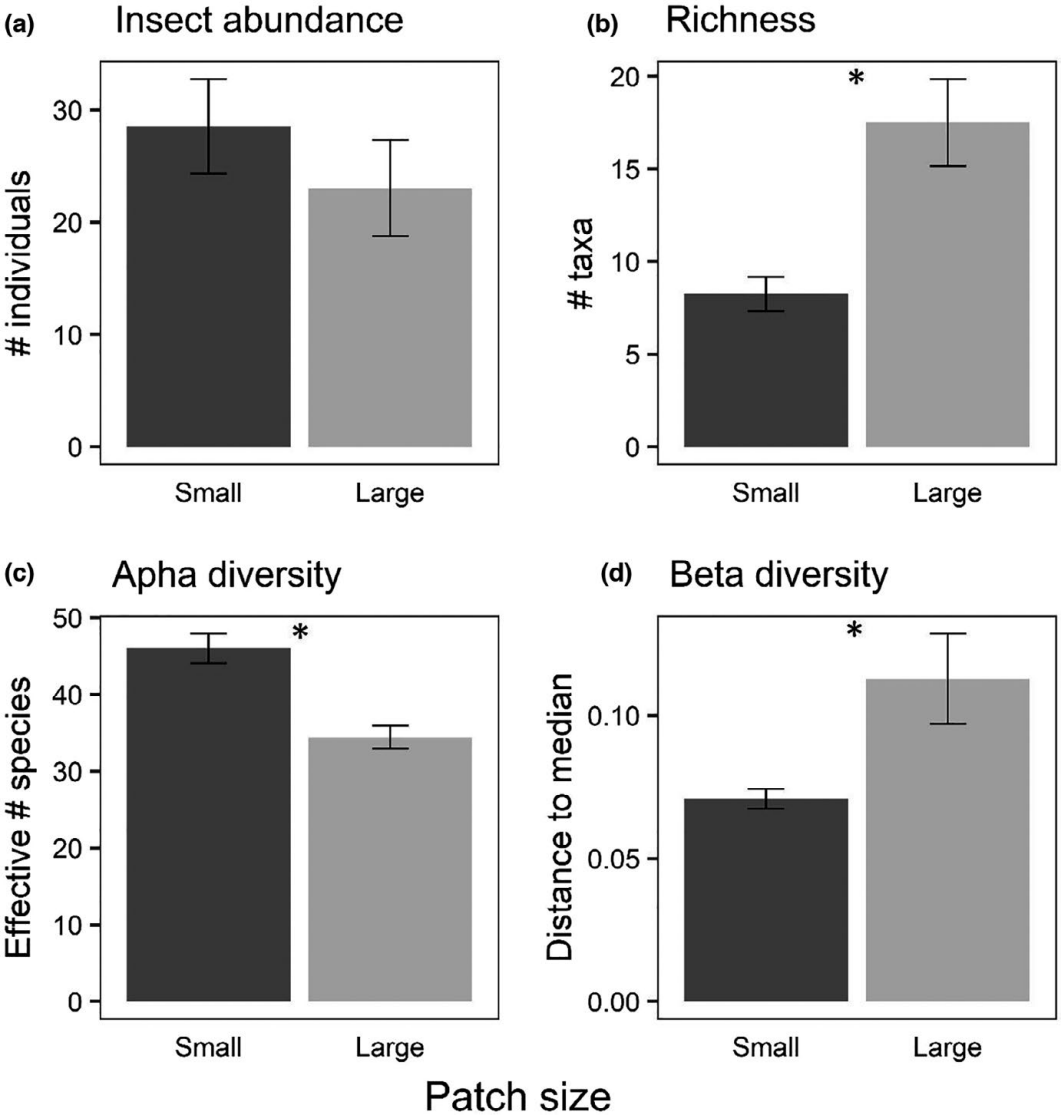
Mean (± *SE*) (a) size‐adjusted abundance of all insects, (b) number of species, (c) alpha diversity (Jost's effective number of species (Jost, 2006)), and (d) beta diversity (distance to median) from the patch size experiment (exp. 2). Asterisks indicate significant differences (*p* < .05) between patch sizes

**FIGURE 7:**
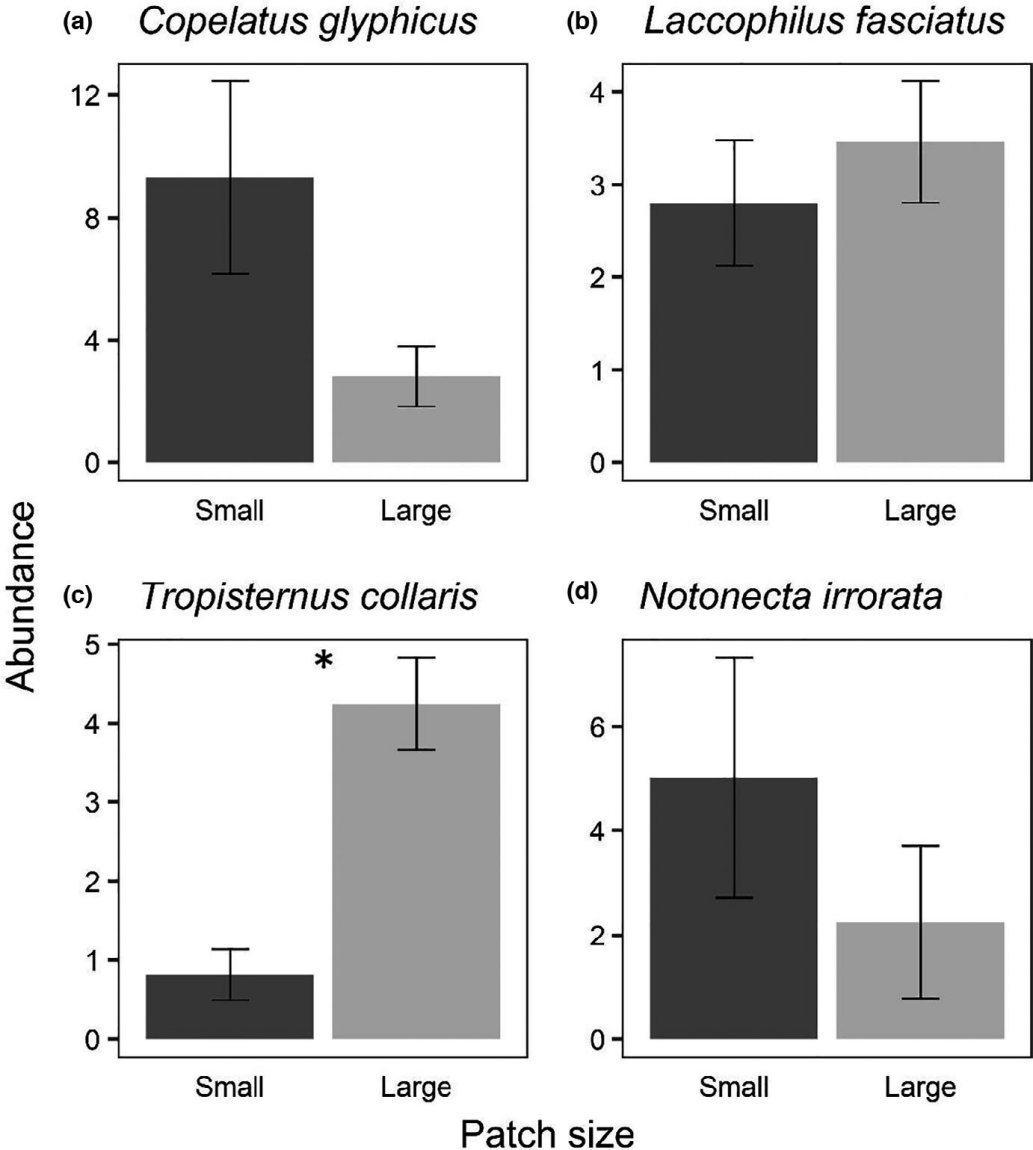
Size‐adjusted average number of colonists (± *SE*) per small and large mesocosm from the patch size experiment (exp. 2) for the four most abundant species (*N* > 100). The asterisk indicates a significant difference (*p* < .05) between patch sizes

**FIGURE 8:**
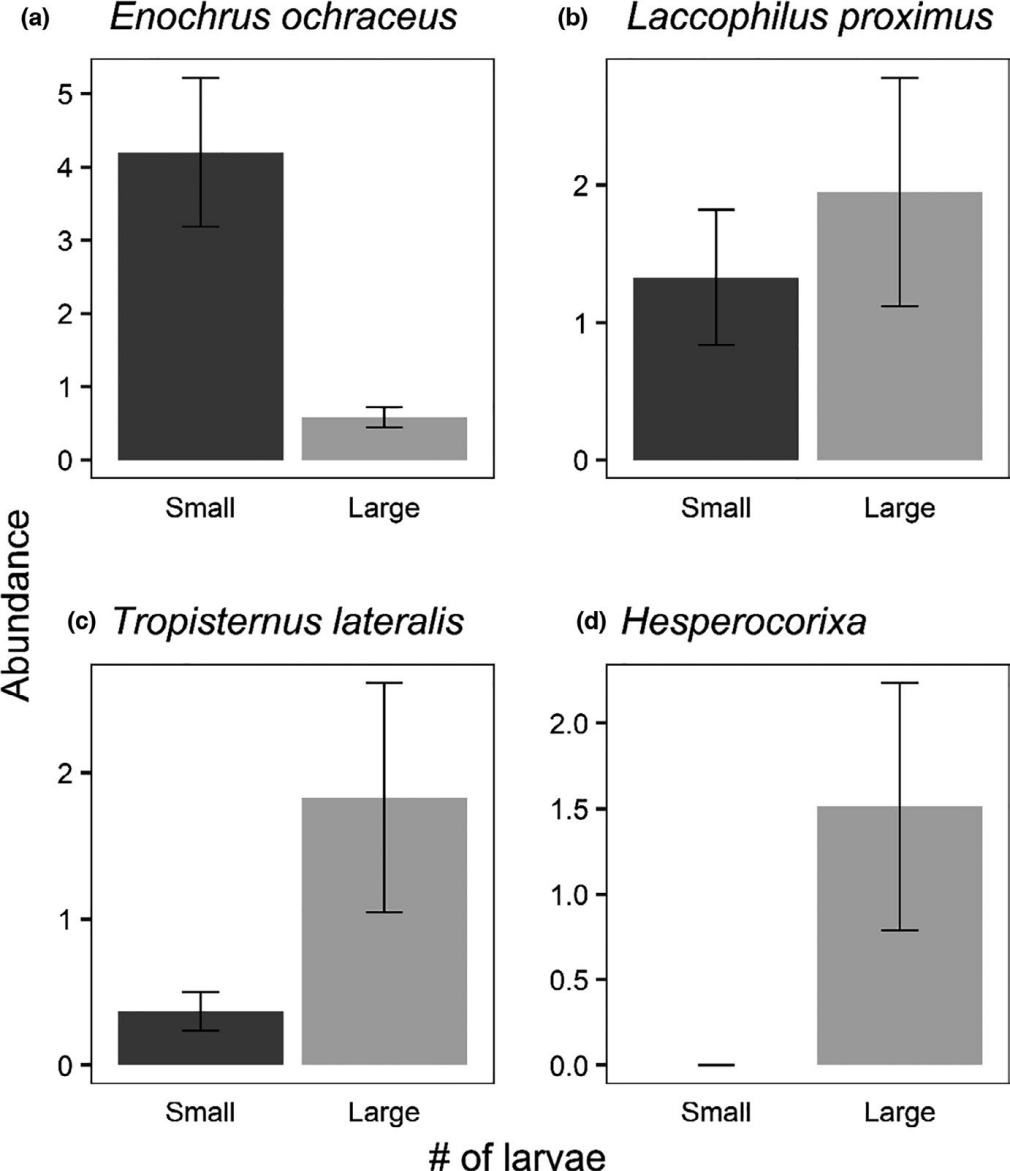
Patch area‐adjusted average number (± *SE*) of colonists per small and large mesocosms from the patch size experiment (exp. 2) for the fifth through eighth‐most abundant taxa (50 < *N* < 100). Statistical tests were not performed for these four species due to a priori limitations on which species we tested individually. However, we present the results here to illustrate the patterns are consistent with prior work (Resetarits et al., 2019) and our experiment 3

**FIGURE 9:**
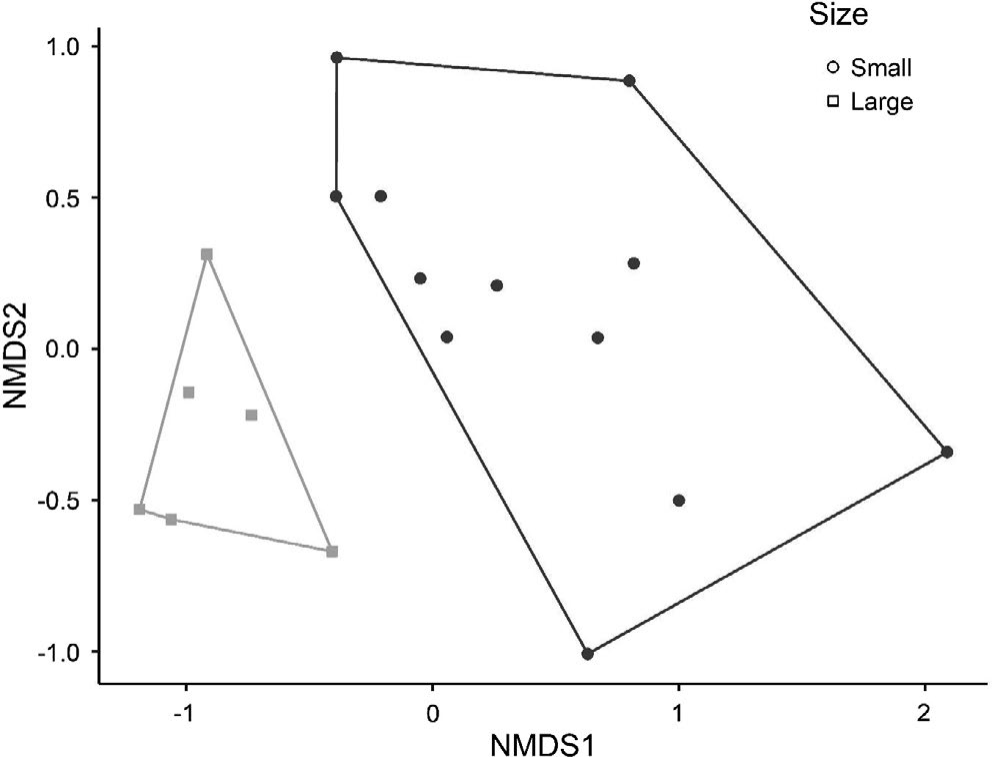
NMDS plot the insect assemblage in the patch size experiment (exp. 2) between large and small mesocosms

A total of 931 beetles representing 29 species/genera in four families (Table 1) and 248 hemipterans representing 10 species/genera in five families colonized the experiment (Table 2). Overall insect abundance did not vary between patch sizes (Table 5, Figure 6a), but there were significant differences in richness, alpha diversity, community structure, and beta diversity between patch sizes. Richness was higher in large patches (Figure 6b), alpha diversity was higher in small patches (Figure 6c), and beta diversity was higher in large patches (Figure 6d). Four species were above our individual analysis threshold (*N* > 100; Figure 7), and only one of these had significant difference in area‐adjusted colonization rates between patches of different sizes: *Tropisternus collaris*, which preferred large patches (Figure 7c). However, both *Copelatus glyphicus* (Figure 7a) and *Notonecta irrorata* (Figure 7d) had clear trends in directions consistent with the size + density experiment (experiment 3) and Resetarits et al. (2019), while the non‐response by *Laccophilus fasciatus* (Figure 7b) was also consistent with these other experiments. We provide figures of the next four most abundant species (Figure 8; 50 < *N* < 100) to illustrate that despite the somewhat different experimental design, we had consistent size preferences by individual species in this experiment as our experiment 3 and that of Resetarits et al. (2019). Assemblage structure was strongly affected by patch size, with distinct assemblages in large and small patches (Table 4, Figure 9).

**TABLE 4:** Analysis results from the patch size experiment (exp. 2). All results are for the effects of patch size, with the exception of insect richness and community structure, which include additional factors (listed individually below) in analyses. $\eta_{P}^{2}$ is an estimate of effect size. Bold indicates significant results (*p* < .05)

Taxon	SS	*df*	*F*	*p*	$\eta_{P}^{2}$
Insect abundance	0.84	1, 15.5	0.6	.47	0.03
Richness					
Abundance	1.68	1, 14.8	12.6	.**0030**	0.45
Size	7.72	1, 11.9	57.7	**<.0001**	0.79
Alpha diversity	555.36	1, 18	17.2	.**0006**	0.49
Community structure					
Size	0.11	1, 11	18.7	.**0001**	0.63
Block	0.08	5, 11	2.5	.**020**	0.54
Beta diversity	0.01	1, 16	12.6	.**0027**	0.44
*Copelatus glyphicus*	4.19	1, 15.6	2.9	.11	0.15
*Laccophilus fasciatus*	0.26	1, 15.6	0.7	.40	0.04
*Notonecta irrorata*	0.90	1, 12.0	1.5	.24	0.10
*Tropisternus collaris*	4.73	1, 12	44.7	**<.0001**	0.75

### 3.3 Experiment 3: Hyla larval density + patch size

**FIGURE 10:**
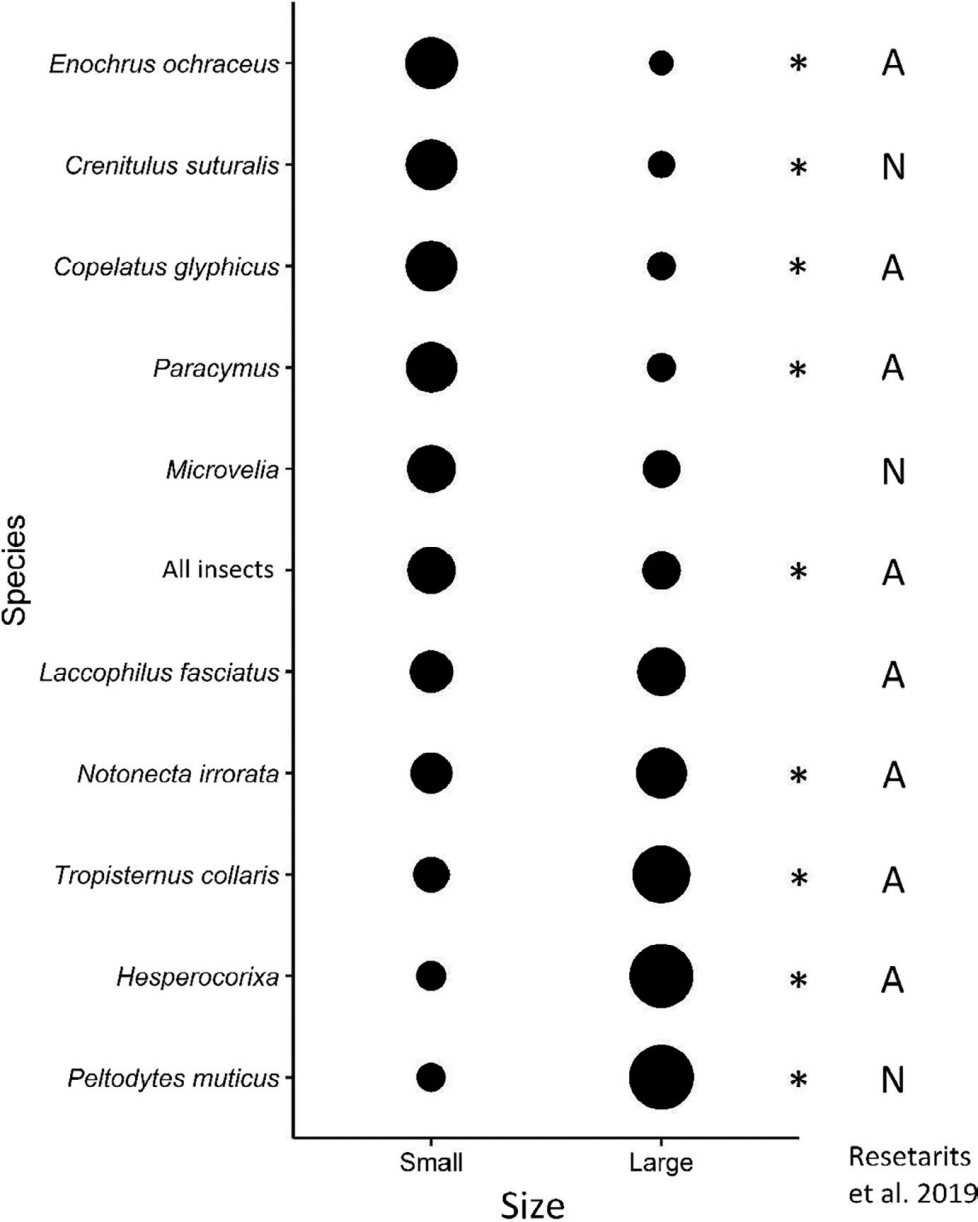
The average area‐adjusted proportion of colonists for all insects and the 10 most abundant taxa per small patch (two patches per block) and large patch (one patch per block) from the *Hyla* larval density + patch size experiment (exp. 3). Taxa are ordered from top to bottom by increasing proportion of individuals within large patches. Asterisks indicate significant differences between patch sizes (*p* < .05). The right column indicates whether responses of each taxa agree (A) with those published in Resetarits et al. (2019) or whether they are newly tested species (N). There were no disagreements in responses by taxa between the two experiments

**FIGURE 11:**
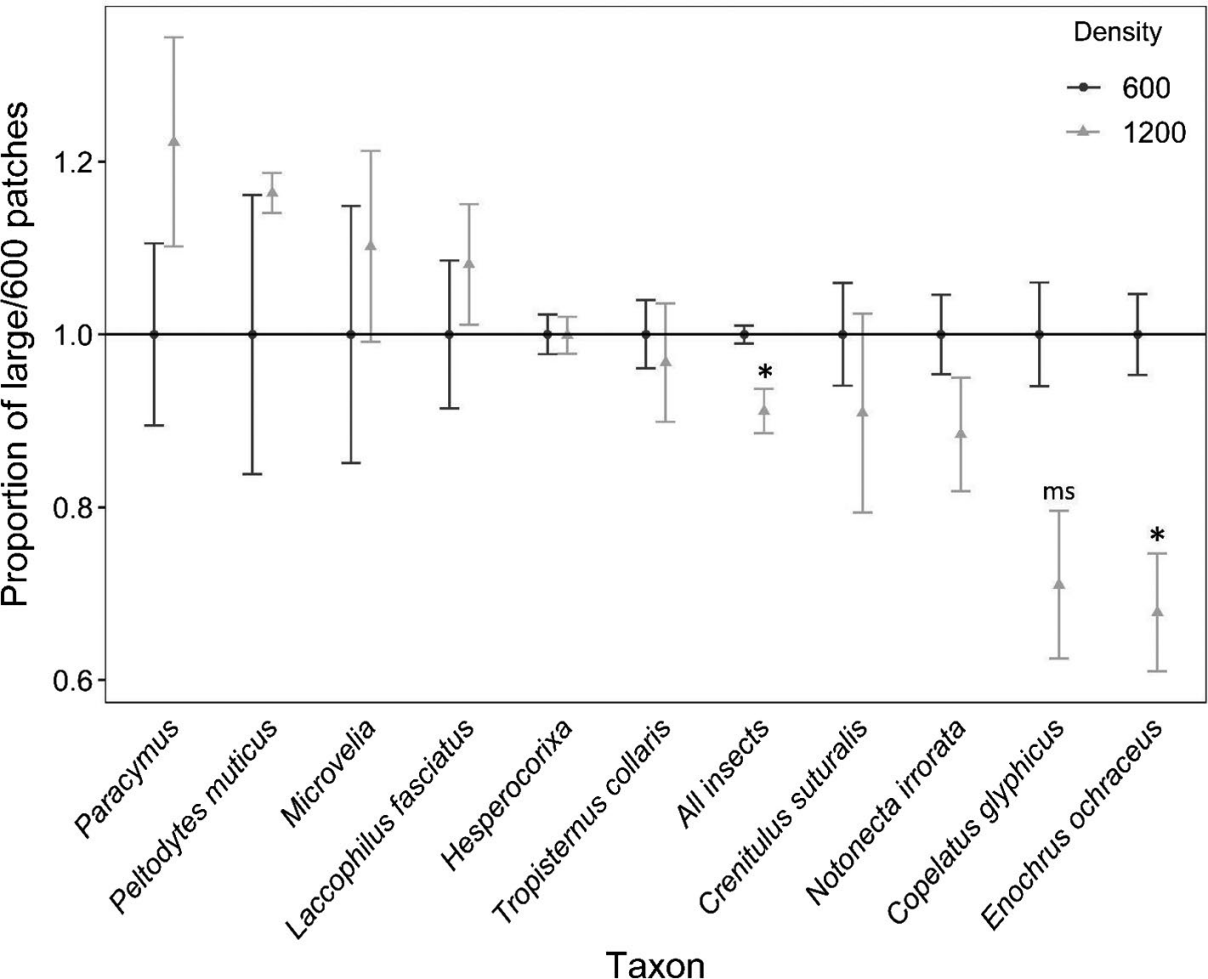
The proportion of colonists (± *SE*) within a block that colonized large mesocosms containing 1200 *H*. *chrysoscelis* larvae (light gray) as a proportion of the average colonization rate of large mesocosms containing 600 larvae (dark gray) for all insects and the 10 most abundant (*N* > 100) taxa from the *Hyla* larval density + patch size experiment (exp. 3). Taxa are ordered left to right by decreasing proportion in large mesocosms from blocks containing 1200 larvae. Asterisks indicates a significant difference (*p* < .05) in proportions between densities; ms indicates a marginal difference (.05 < *p* < .10)

**FIGURE 12:**
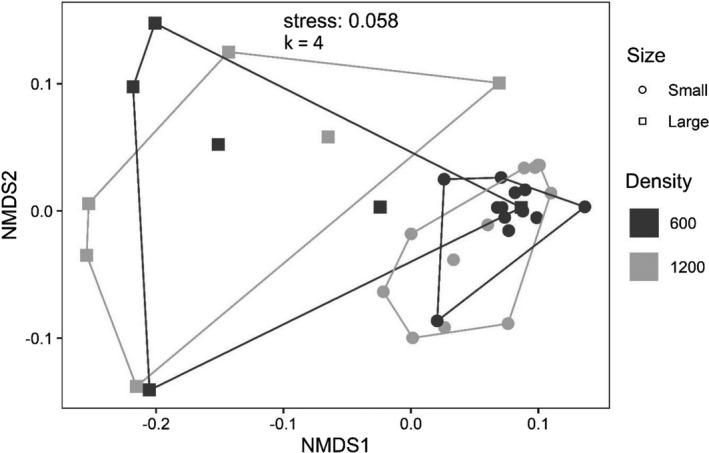
NMDS plot of the insect assemblage across patch size and density of *H*. *chrysoscelis* larvae in large mesocosms (locality‐level treatment) from the *Hyla* larval density + patch size experiment (exp. 3)

A total of 4076 beetles representing 45 species/genera in six families (Table 1) and 1500 hemipterans representing 13 species/genera in six families colonized the experiment (Table 2). Patch size had numerous and relatively large effects in community (Table 5) and most abundance (Table 6) analyses, while *Hyla* density had few effects. Total insect abundances were higher in small patches (Figure 10, “All insects”), while the proportion of all insects in large mesocosms was lower in those containing 1200 larvae than those with 1600 larvae (Figure 11, “All insects”). Richness strongly positively covaried with abundance (Table 5a), and large mesocosms (23.3 ± 1.6 species; mean ± *SE*) had more species than small mesocosms (13.0 ± 0.9 species). The proportion of taxa within a block that colonized large mesocosms was marginally higher in localities with 600 larvae (0.64 ± 0.01) than those with 1200 larvae (0.58 ± 0.03), while the proportion that colonized small mesocosms did not vary with *Hyla* density (Table 5c). Alpha diversity was higher in small mesocosms (35.9 ± 1.2 effective number of species) than large (28.2 ± 1.1 effective number of species), but was unaffected by *Hyla* density (Table 5a). Beta diversity was higher in large mesocosms (0.16 ± 0.01 distance to median) than small mesocosms (0.11 ± 0.01), higher in small mescososms in localities with 1200 larvae (0.12 ± 0.01) than small mesocosms with 600 larvae (0.09 ± 0.01), but did not vary between large mesocosms based on *Hyla* density (Table 5a). Patch size generated unique assemblage structures between large and small mesocosms (Figure 12), while assemblages were marginally different among small mesocosms within localities containing 600 versus 1200 *Hyla* larvae, but assemblage structure did not vary among large mesocosms based on *Hyla* density (Table 5b). However, the marginal difference among small mesocosms is likely due to PERMANOVA’s inability to distinguish location versus dispersion effects, which is supported by the similarly‐significant beta diversity results among small mesocosms with a larger effect size (Anderson & Walsh, 2013).

**TABLE 5:** Results of assemblage analyses in the *Hyla* larval density + patch size experiment (exp. 3). Results in (a) for richness and alpha diversity are a single analysis on all factors listed; analyses for beta diversity are separate analyses for each row (see methods for details). Results in (b) are for assemblage structure using PERMANOVA. Results in (c) for richness are two separate logistic regressions for the proportion of taxa among large and small mesocosms between localities of different densities. $\eta_{P}^{2}$ is an estimate of effect size. Bold indicates significant results (*p* < .05); italics indicates marginal results (.05 < *p* < .10)

(a)	SS	*df*	*F*	*p*	$\eta_{P}^{2}$
Richness					
Abundance	2.3	1, 36	12.4	.**0012**	0.14
Size	14.0	1, 34	75.7	**<.0001**	0.69
Alpha diversity					
Size	471.9	1, 31.9	22.1	**<.0001**	0.40
Density	17.7	1, 31.9	0.8	.37	0.02
Beta diversity					
Size	0.024	1, 34	24.6	**<.0001**	0.42
Density w/in L	0.002	1, 10	1.2	.30	0.11
Density w/in S	0.003	1, 22	4.8	.**039**	0.18

(b) Assemblage structure	SS	*df*	*F*	*p*	
Size					
Size	0.30	1	23.9	**<.0001**	
Location	0.15	2	6.1	**<.0001**	
Round	0.10	1	18.4	**<.0001**	
Density within large					
Density	0.02	1	1.9	.30	
Location	0.13	2	4.3	.**0003**	
Round	0.08	1	4.8	.**0014**	
Density within small					
Density	0.02	1	1.8	*.07*	
Location	0.07	2	4.1	.**0001**	
Round	0.06	1	7.4	.**0001**	

(c) Richness		*df*	χ^2^	*p*	
Density w/in L		1, 11	2.8	.095	
Density w/in S		1, 11	1.8	.18	

**TABLE 6:** Analysis of overall insect abundance and abundance of individual taxa in the *Hyla* larval density + patch size experiment (exp. 3). Separate analyses were conducted for the effects of size (area‐adjusted colonization) and density (proportion of colonists in large mesocosms; logistic regression). $\eta_{P}^{2}$ ‐ is an estimate of effect size. Bold indicates significant results (*p* < .05), and italics indicates marginal results (.05 < *p* < .10)

	Size					Density	
	SS	*df*	*F*	*p*	$\eta_{P}^{2}$	χ^2^	*p*
All insects	22.29	1, 32	14.2	.**0007**	0.30	3.88	.**049**
*Copelatus glyphicus*	31.75	1, 32	16.6	.**0003**	0.33	3.29	.*070*
*Crenitulus suturalis*	16.27	1, 32	8.8	.**0056**	0.21	0.09	.76
*Enochrus ochraceus*	21.68	1, 36	16.2	.**0003**	0.31	5.07	.**024**
*Hesperocorixa*	10.76	1, 32	19.6	**.0001**	0.37	0.92	.34
*Laccophilus fasciatus*	1.96	1, 30	2.1	.15	0.06	1.16	.28
*Microvelia*	1.27	1, 32	1.7	.20	0.05	0.47	.49
*Notonecta irrorata*	2.58	1, 32	5.3	.**028**	0.14	1.46	.23
*Paracymus*	5.53	1, 33	8.9	.**0054**	0.21	0.06	.80
*Peltodytes muticus*	5.52	1, 33	40.6	**<.0001**	0.55	1.21	.27
*Tropisternus collaris*	2.49	1, 32	8.1	.**0076**	0.20	0.07	.79

There was considerable taxon‐specific variation among insects in patch area‐adjusted colonization rates. Among the abundant taxa (*N* > 100), four taxa colonized smaller patches at higher rates than large patches: the dytiscid *Copelatus glyphicus* and three hydrophilid taxa, *Enochrus ochraceus*, *Crenitulus suturalis*, and *Paracymus* spp. (Table 6; Figure 10). There were four taxa colonized large patches at higher rates than small patches: the hydrophilid *Tropisternus collaris*, the haliplid *Peltodytes muticus*, the notonectid *Notonecta irrorata*, and the corixid genus *Hesperocorixa*. Two taxa had no significant difference in colonization rates among patch sizes: the veliid genus *Microvelia* and the dytiscid *Laccophilus fasciatus*. The density of *Hyla* larvae in large patches did not affect the proportion of colonists within blocks that colonized large mesocosms for eight of the ten common taxa (Table 6, Figure 11). One species, *E*. *ochraceus*, had a significantly lower proportion of colonists in large mesocosms (and higher proportion in small mesocosms) when large mesocosms contained 1200 larvae than there were in large mesocosms when large mesocosms contained 600 larvae. Similarly, there was a marginally lower proportion of *C*. *glyphicus* in large mesocosms containing 1200 larvae than when large mesocosms contained 600 larvae (Figure 11).

## 4 DISCUSSION

Immigration rates are one of the primary determinants of the abundance and distribution of species at multiple spatial scales, and the order of species arrival can affect colonization rates of later‐arriving taxa through priority effects. We directly manipulated one of the most well‐documented determinants of species distribution patterns, patch size, both independently and concurrently with variation in the presence and density of larval *H*. *chrysoscelis*, one of the most abundant and quickly colonizing species in fishless lentic habitats of the southeastern United States. Overall, we observed that *Hyla* larvae had few independent effects on colonization, and effects on assemblages were limited to shifting more colonists toward small mesocosms when near large mesocosms with high *Hyla* densities. Conversely, patch size had numerous, strong, and functionally diverse effects on colonizing insect taxa (Resetarits et al., 2019). Both factors are important for understanding the abundance, distributions, and niches of aquatic insects.

Larval *Hyla* density had almost no effect on insect assemblages or colonization of individual taxa. Many of these taxa could be predators or competitors of *Hyla* larvae, but the lack of insect responses suggests that either they are not predators of *Hyla* larvae or, perhaps more likely, that the abundance and distribution of this single potential prey species are not important factors in habitat selection. This lack of response could be a lack of avoidance/attraction or a lack of detection, a difference that we cannot separate here. The results of this experiment suggest that the role of *Hyla* larvae in colonization by aquatic insects is minimal. When the roles are reversed, presence of beetles within patches deters oviposition by adult *Hyla* (Pintar & Resetarits, 2020c). Thus, colonization decisions and distribution patterns among species in this predator–prey interaction are driven primarily by the need for ovipositing *Hyla* to avoid insect predation, but not by colonizing insects optimizing foraging opportunities based on a single prey species.

Lack of significant effects of patch size on colonization by aggregate groups in our patch size experiment (exp. 2) was likely due to both lower abundances of individual species and variation in responses by species that composed aggregate groups. Common species with similar abundances, like *T*. *collaris* and *E*. *ochraceus* (both Hydrophilidae), had contrasting individual responses, resulting in non‐significant differences overall. These differences between experiments occurred because this size experiment was conducted later in the summer than the size + density experiment (main text) or the study by Resetarits et al. (2019), and the abundance, richness, and assemblage composition of dispersing insects are typically lower and different later in the summer, but our results here do not necessarily conflict with either of those experiments at the species level. However, for community metrics, species richness, alpha diversity, beta diversity, and assemblage structure all had clear, relatively strong effects consistent with prior work.

Combining variation in *Hyla* density with patch size illustrated the strong effects that patch size has on insect colonization (Figure 10), but some results were unexpected based on independent tests of *Hyla* density and patch size. There were taxon‐specific preferences for both large and small patches, as well as taxa with no response to patch size (Figure 10). These taxon‐specific responses generated unique assemblage structures between patch sizes (Figure 12), with large patches having higher richness, lower alpha diversity, and higher beta diversity. Despite a somewhat different experimental design than Resetarits et al. (2019), we observed many of the same taxon‐specific patch size preferences. Here, we add that *C*. *suturalis* prefers small patches, and *P*. *muticus*, the first haliplid tested, prefers larger patches (Figure 10). No other beetles have exhibited such a strong preference for large patches as *P*. *muticus*, and despite their smaller size, haliplids have morphological characteristics that make them remarkably resistant to predation (Pintar & Resetarits, 2021), potentially enabling them to persist with fish or other predators common larger ponds. We also observed the first response, or rather non‐response, by a semiaquatic hemipteran (Gerromorpha), *Microvelia* spp.

Although our design of experiment 3 confounds the presence/absence of *Hyla* larvae with patch size, it is clear that differences in colonization in this experiment are largely due to patch size. Our experiment that directly manipulated *Hyla* larval density (exp. 1) showed almost no responses by colonizing insects to *Hyla* larval density, while the proportion of colonists that were shifted among mesocosms of different size in response to variation in *Hyla* larval density (exp. 3) also varied among few taxa. In contrast, patch size exhibited strong effects and consistent independent patterns in our experiment 2 and the experiment by Resetarits et al. (2019) as we observed in our experiment 3. We had many of the same species in each of these experiments, so we would not expect the independent non‐responses to *Hyla* larval density and strong responses to patch size to suddenly change when both patch characteristics are combined into a single experiment. Hence, we believe that, despite the confounding of patch size and presence/absence of *Hyla* larvae in experiment 3, the effects we observed in patch size analyses in experiment 3 are indeed indicative of patch size effects on colonization and not effects of the presence of *Hyla* larvae.

For colonizing insects, patch size continues to be one of the most dominant factors driving colonization rates across experimental landscapes, rivaled and exceeded perhaps only by the threat of predation posed by fish (Resetarits et al., 2019, 2021). In the absence of a direct response to a species, habitat selection based on patch size could be an indirect way of responding to species typically found in patches of certain sizes, like the higher probability of finding fish in larger, permanent ponds and the strong preference by *Hyla* to oviposit in larger patches (Resetarits et al., 2018). So it is possible the colonizing individuals could use patch size as a proxy to determine the probability that *Hyla* is present. However, most of our abundant insect taxa here are known to avoid fish (Resetarits & Pintar, 2016; Resetarits et al., 2019) but largely do not respond directly to *Hyla* density. So if the variation in patch size preferences by our insects here is a proxy for detection of another species, it almost certainly being used to avoid predatory fish, or other effective predators that prefer larger patches (e.g., *Notonecta irrorata* or the largest dytiscids and hemipterans that rarely colonize even our largest mesocosms).

Although the effects of patch size in this study were numerous, larval *Hyla* density shaped assemblages but only had two species‐specific effects, both on species that strongly prefer small patches. In all cases, the proportion of colonists that colonized large mesocosms was higher in blocks with 600 larvae than with 1200 larvae. For *E*. *ochraceus* and *C*. *glyphicus*, this suggests that higher densities of *Hyla* larvae in large mesocosms further shifted colonization away from large patches and to small patches that these species already preferred. For the insect assemblage, this resulted in not only a smaller proportion of colonists in large patches containing 1200 larvae than 600 (Figure 11), but also a marginally lower proportion of total taxa (64% with 600 larvae, 58% with 1200 larvae). A greater proportion of colonists in small mesocosms in localities with 1200 larvae did not result in higher richness or alpha diversity in small mesocosms, but it did result in higher beta diversity, indicating greater variation in assemblage structure. While our analysis of assemblage structure (Table 5b) indicated significant differences among small mesocosms based on *Hyla* density, the high spatial overlap in NMDS (Figure 12) suggests this might be an indication of the significant variation in distance to median (beta diversity, Table 5a) rather than significant differences in assemblage structures. Overall, these results of *Hyla* density support the idea that higher *Hyla* larvae densities in large mesocosms shift some of the colonization from large mesocosms to small.

Our results suggest that abundance of *Hyla* larvae in ponds can impact colonization by aquatic insects in ways that are largely observed at the assemblage (community) scale. The proportion of individuals and proportion of species were both higher in large patches containing fewer (600) *Hyla* larvae. These patterns suggest a slightly dominant, competitive role for *Hyla* larvae, wherein through their own strong preference for oviposition in large patches (Resetarits et al., 2018) can alter the distribution of species among patches that are part of the same metacommunity. Dispersal in aquatic insects is well documented (Johnson, 1969), and aquatic insects are capable of traveling long distances via flight. However, dispersal in the context of this experiment refers to the rate at which individuals move into and between sites (Heino et al., 2015). In this respect, dispersal by aquatic insects is limited, as once colonization of a patch occurs secondary dispersal and colonization is rare (Zera & Denno, 1997). However, spatial context dependence is an important consideration for the colonization of aquatic insects (Pintar & Resetarits, 2017d), with processes driving colonization at a larger scale differing from those at smaller scales. Hence, *Hyla* larvae likely have few effects on colonists arriving into localities, but do among patches within localities in conjunction with the effects of patch size on most taxa. In this sense, the effects of *Hyla* larvae are through the redistribution of individuals among patches during this initial colonization process. Here, our focus was on effects of *Hyla* larvae on primary colonization by aquatic insects, which combined with the short time to metamorphosis necessitated short duration experimental rounds of our experiments. It is possible that effects of *Hyla* on aquatic insect assemblages can be greater than observed here if communities were allowed to develop further (Vonesh et al., 2009).

Variation in both patch size and *Hyla* larval density together influenced assemblage structure of aquatic insects. Although patch size clearly plays an important role in colonization by a variety of organisms, the reasons for these preferences remain unknown (Resetarits et al., 2019). A tempting answer is that pond size increases hydroperiod and thereby makes larger patches a more stable environment, and previous research has shown that hydroperiod is an important factor in affecting anuran assemblages (Eason & Fauth, 2001; Rowe & Dunson, 1993; Wilbur, 1987). However, when examining hydroperiod and patch size separately, hydroperiod accounts for most variation in species richness, with patch size playing a relatively small role (Babbitt, 2005; Semlitsch & Bodie, 1998). Additionally, patch size has been shown to have a relatively weak effect on hydroperiod, with other factors such as depth (which did not vary in this experiment), vegetation, and underlying hydrology playing more important roles in determining hydroperiod (Eason & Fauth, 2001; Snodgrass et al., 2000). Similarly, traits of aquatic insects, such as larval period length, feeding habits, resistance to predation, among countless others, may play into preferences for patches of different size as they may relate to correlated environmental characteristics.

While other processes may drive structuring of communities post‐colonization (Zhao et al., 2020), habitat selection is the first filter determining which species occupy patches. Although we found few effects when testing *Hyla* larvae alone, larval density in conjunction with patch size had significant impacts on species composition and assemblage structure. Characteristics of habitat patches are clearly important determinants of the abundance and distribution of species across landscapes (Chesson, 2000). The behavioral process of habitat selection during the colonization stage is the initial determinant of distribution and abundance, which are later modified through post‐colonization processes. Historically, patch size was one of the fundamental underlying drivers of species abundances and distributions, and we have built on recent evidence that effects of patch size go beyond extinction and passive capture rates to include behavioral preferences based on patch size, preferences that can be modified at multiple spatial scales by priority effects.

## CONFLICT OF INTEREST

The authors declare no conflicts of interest.

## AUTHOR CONTRIBUTIONS

**Reed C. Scott Jr.:** Conceptualization (lead); Funding acquisition (equal); Investigation (lead); Methodology (lead); Writing‐original draft (equal); Writing‐review & editing (equal). **Matthew R. Pintar:** Data curation (lead); Investigation (supporting); Methodology (supporting); Visualization (lead); Writing‐original draft (equal); Writing‐review & editing (equal). **William J. Resetarits Jr**.**:** Conceptualization (supporting); Funding acquisition (equal); Methodology (supporting); Supervision (lead); Writing‐original draft (supporting); Writing‐review & editing (supporting).

## Data Availability

Data are available in Dryad at https://doi.org/10.5061/dryad.gxd2547nj
