# Corrigendum

**DOI:** 10.1002/ece3.8928

**Published:** 2022-05-15

In the recent article by Scott et al. (2021), it has come to the authors’ attention that the addition of a constant during square‐root transformation of the species matrices (√(X+0.5)) produced incorrect results for analyses of three assemblage metrics: alpha diversity, beta diversity, and assemblage structure. The authors have corrected these analyses by square‐root transforming community matrices without the constant added (√X). The authors apologize for this oversight. Corrected R code is posted on Dryad at https://doi.org/10.5061/dryad.gxd2547nj

This error was due to the conversion of all zero abundances of species (absence) to positive values (presence). Although the addition of the constant is appropriate for univariate analyses of abundances and richness, the effects on community analyses were particularly notable for alpha diversity (effective number of species).

## 1 EXPERIMENT 1: *HYLA* LARVAL DENSITY

Initial analyses indicated a marginal effect of *Hyla* larval density on alpha diversity, whereas revised analyses indicate no effect. Both initial and revised analyses show that there was no effect of *Hyla* larval density on either assemblage structure or beta diversity. Overall, revised analyses continue to support or initial conclusion that *Hyla* larvae have little to no effect on aquatic insect colonization and community structure.

Corrected analysis results of alpha diversity, assemblage structure, and beta diversity from Experiment 1 in Table 3. Bold indicates significant results (*p* < .05)

**Table:** 

	SS	*df*	*F*	*p*	$\eta_{P}^{2}$
Alpha diversity	23.57	4, 39.9	1.1	.35	0.10
Assemblage structure (PERMANOVA)					
Density	0.47	4, 36	1.1	.28	0.11
Location	1.89	2, 36	9.2	.**0001**	0.34
Round	1.66	2, 36	8.1	.**0001**	0.31
Beta diversity	0.02	4, 40	0.6	.64	0.06

Corrected graphs of alpha diversity (Figure 3c) and beta diversity (Figure 3d) from Experiment 1. There were no differences among treatments.

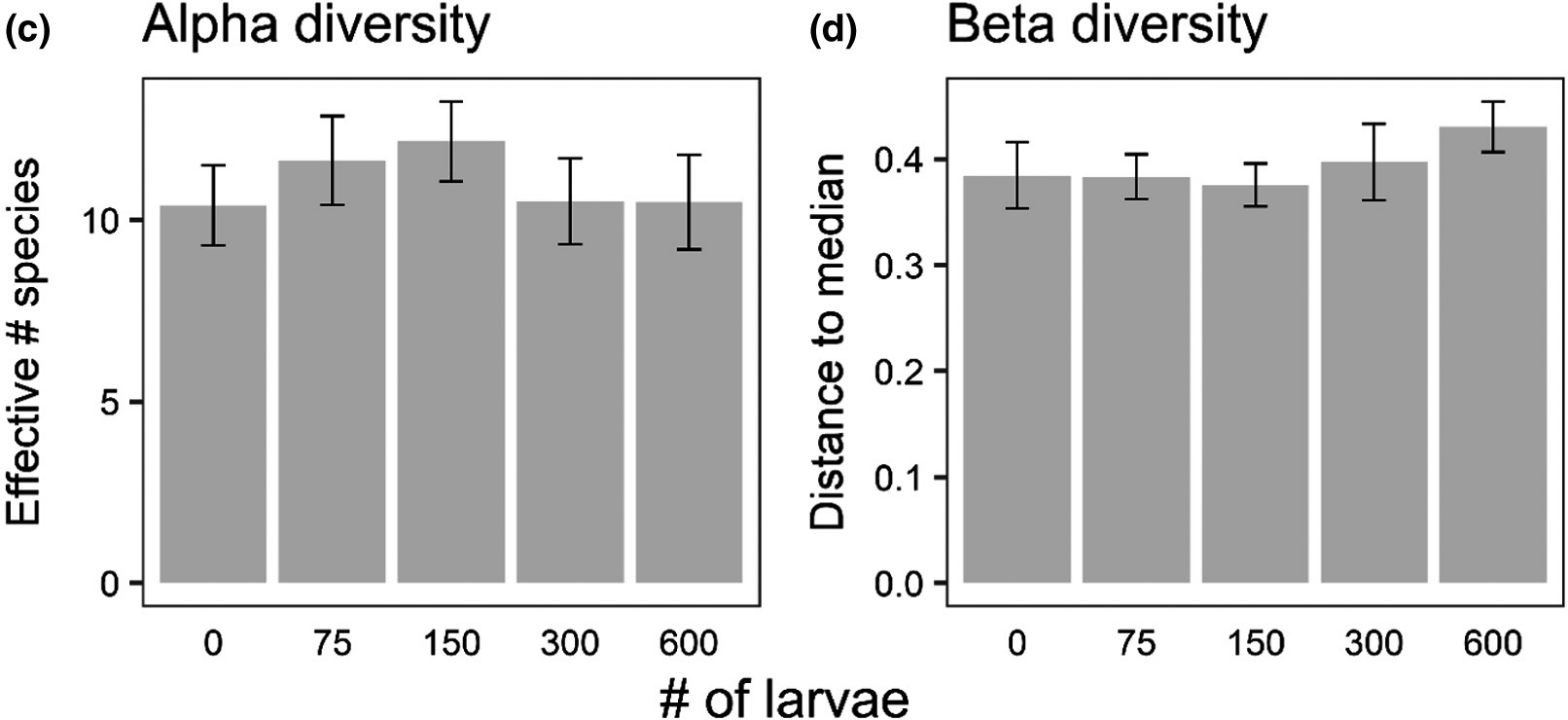

Corrected NMDS plot of assemblage structure in Experiment 1 (Figure 5). There were no differences among treatments.

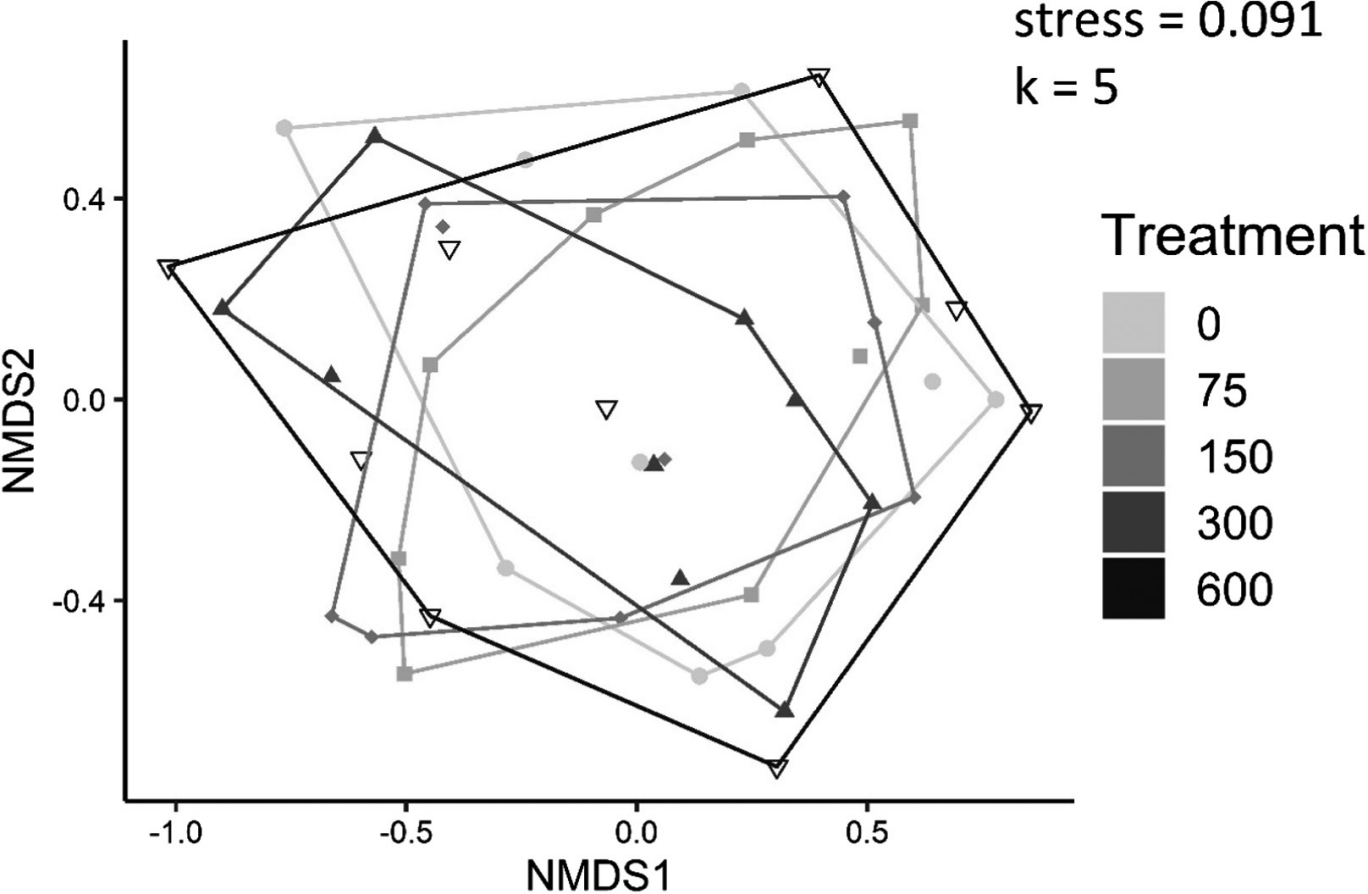

## 2 EXPERIMENT 2: PATCH SIZE

Initial analyses indicated significantly higher alpha diversity in small patches, whereas corrected analyses show that alpha diversity was higher in large patches. Similarly, initial analyses indicated higher beta diversity in large patches, whereas corrected analyses show that beta diversity was higher in small patches. Overall, revised analyses continue to support our conclusions that patch size has large effects on colonizing insect assemblages.

Corrected analysis results of alpha diversity, assemblage structure, and beta diversity from Experiment 2 in Table 4. Bold indicates significant results (*p* < .05).

**Table:** 

	SS	*df*	*F*	*p*	$\eta_{P}^{2}$
Alpha diversity	137.2	1, 12	20.8	.**0007**	0.59
Assemblage structure (PERMANOVA)					
Size	0.74	1, 11	6.4	.**0001**	0.37
Block	1.15	5, 11	2.0	.**0052**	0.47
Beta diversity	0.06	1, 16	4.7	.**046**	0.23

Corrected graphs of alpha diversity (Figure 6c) and beta diversity (Figure 6d) from Experiment 2. Asterisks indicate statistically significant differences between patch sizes (*p* < .05).

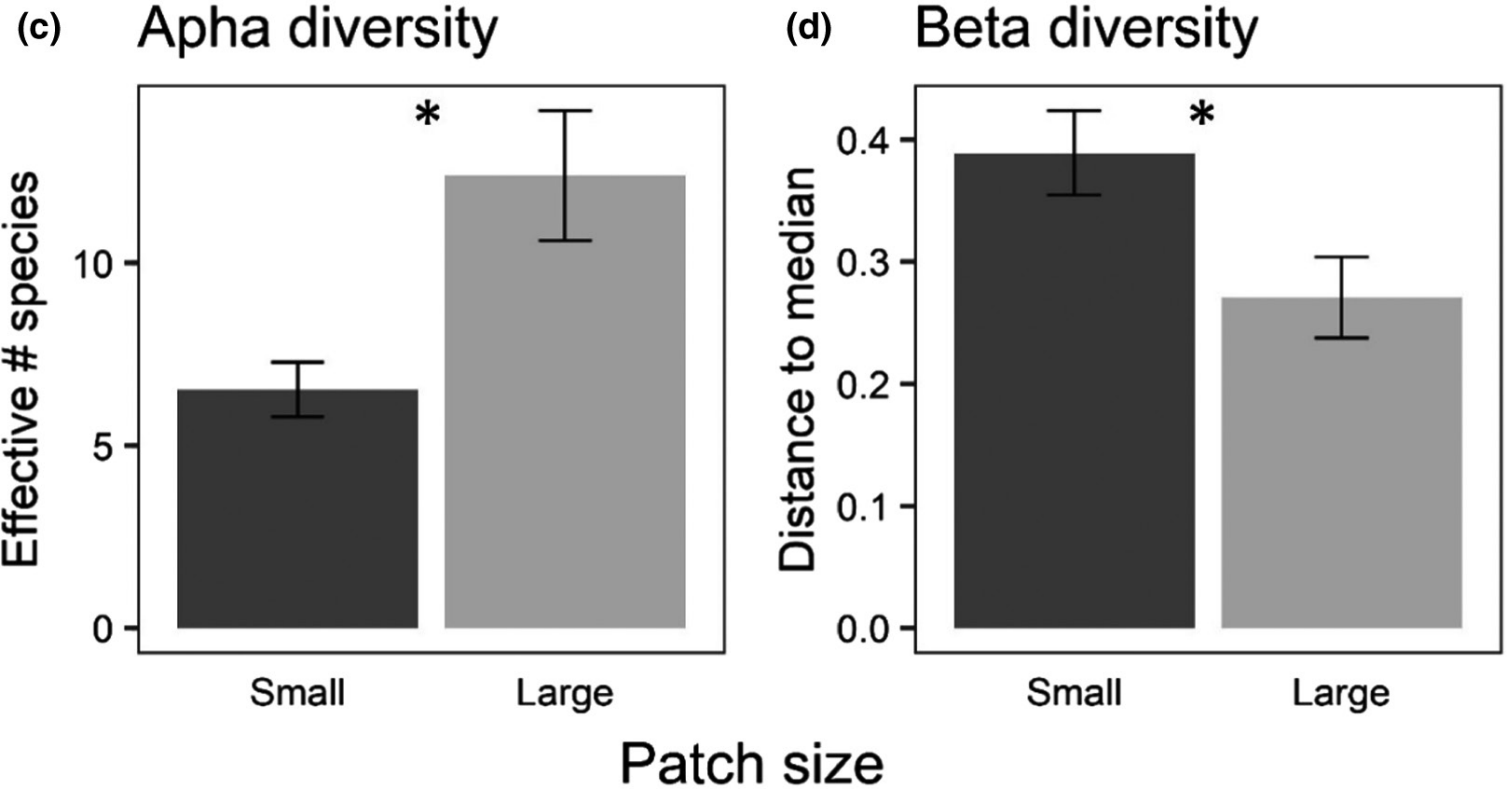

Corrected NMDS plot of assemblage structure in Experiment 2 (Figure 9) between small and large patches.

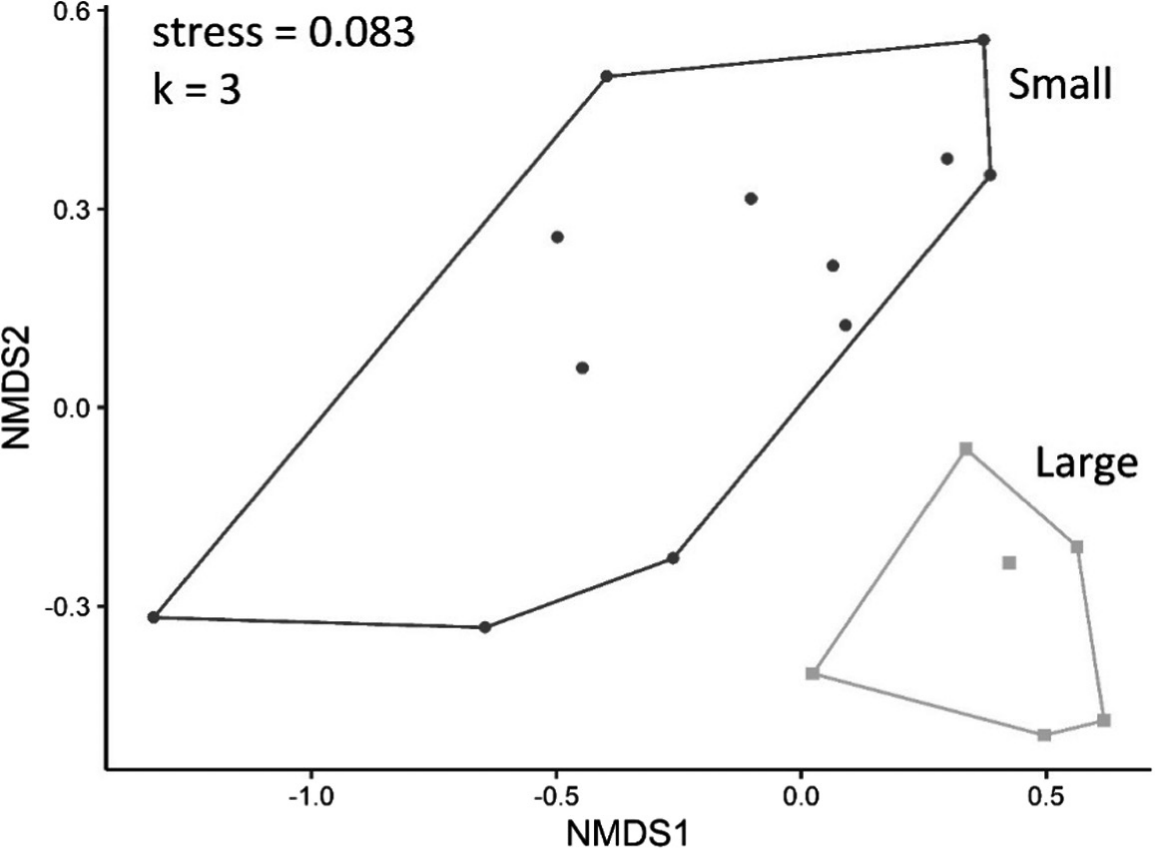

## 3 EXPERIMENT 3: *HYLA* LARVAL DENSITY + PATCH SIZE

Initial analyses indicated large effects of patch size on alpha diversity (smaller mesocosms had a higher number of effective species) and no effect of *Hyla* larval density. Corrected analyses show that large patches had a significantly higher effective number of species (14.2 ± 1.1; mean ± SE) than small patches (9.2 ± 0.7), and patches in localities with 600 *Hyla* larvae had a lower effective number of species (9.8 ± 0.9) than those with 1200 larvae (11.8 ± 4.3). Initial analyses indicated that beta diversity significantly varied with patch size, but that is not supported here. Initial results indicated beta diversity did not vary with patch size based on the density of *Hyla* larvae in those localities; those results are supported here.

Corrected analyses of assemblage structure support initial results. Assemblage structure varied significantly with patch size, did not vary among large mesocosms based on the density of *Hyla* larvae, but did significantly vary between small mesocosms based on the density of *Hyla* larvae (marginally non‐significant in initial analyses). Overall, our results continue to support our initial conclusions that patch size has large effects on colonizing insects, while *Hyla* larval density has limited effects.

Corrected analysis results of alpha diversity, assemblage structure, and beta diversity from Experiment 3 in Table 5. Bold indicates significant results (*p* < .05)

**Table:** 

	SS	*df*	*F*	*p*	$\eta_{P}^{2}$
*(a)*					
Alpha diversity					
Size	199.2	1, 31.5	28.6	**<.0001**	0.47
Density	36.0	1, 31.5	5.2	.**030**	0.14
Beta diversity					
Size	0.012	1, 34	1.7	.20	0.05
Density w/in L	0.007	1, 10	0.8	.40	0.07
Density w/in S	0.006	1, 22	0.9	.36	0.04
*(b) Assemblage structure (PERMANOVA)*					
Size					
Size	1.25	1, 31	14.9	**<.0001**	0.32
Location	0.84	2, 31	5.0	**<.0001**	0.24
Round	0.56	1, 31	6.7	**<.0001**	0.18
Density within large					
Density	0.06	1, 7	1.2	.32	0.14
Location	0.45	2, 7	4.1	.**0002**	0.54
Round	0.24	1, 7	4.5	.**0012**	0.39
Density within small					
Density	0.17	1, 19	2.1	.**028**	0.10
Location	0.64	2, 19	3.9	.**0001**	0.29
Round	0.50	1, 19	6.1	.**0001**	0.24

Corrected NMDS plot of assemblage structure in Experiment 3 (Figure 12).

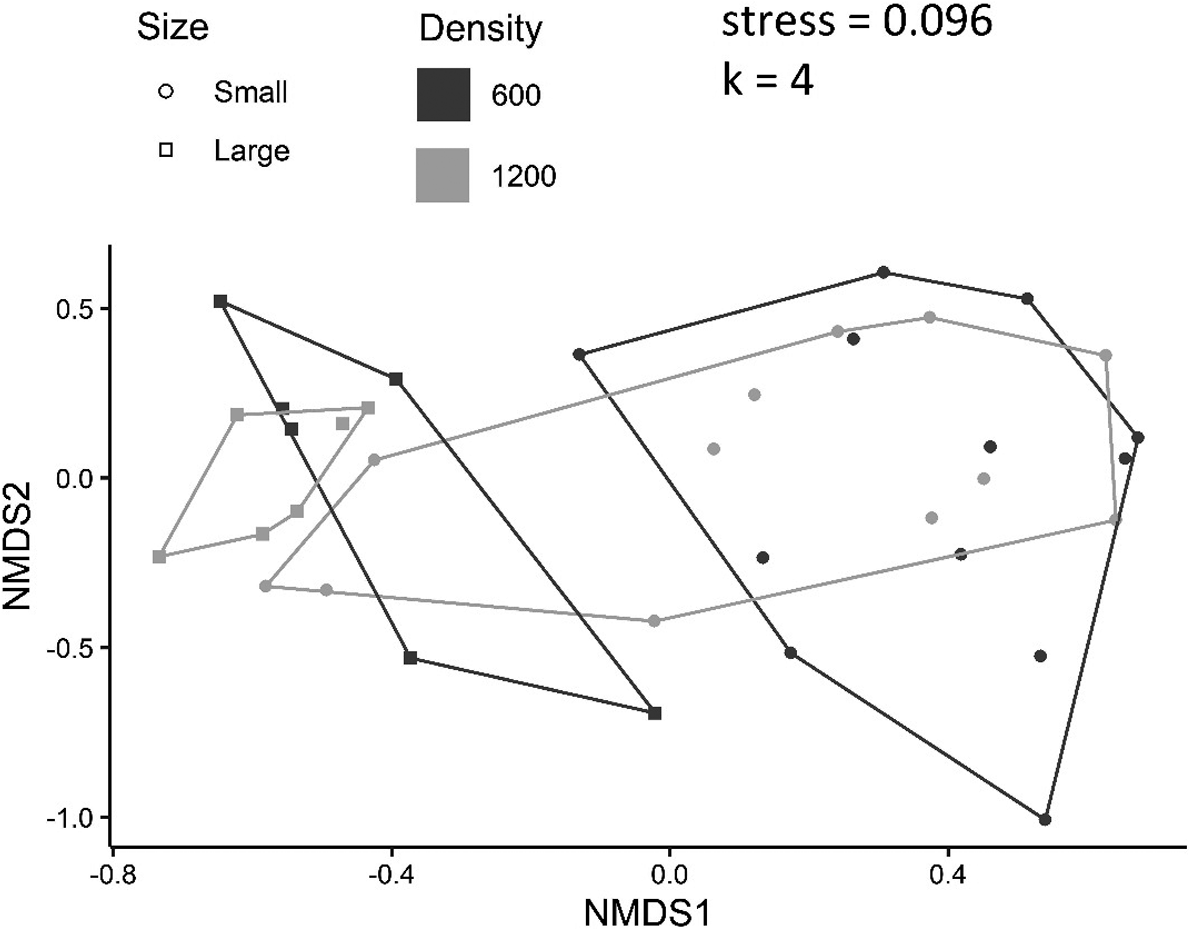

The authors apologize for these errors.
